# Deconstructing heterogeneity of replicative senescence in human mesenchymal stem cells at single cell resolution

**DOI:** 10.1007/s11357-023-00829-y

**Published:** 2023-06-14

**Authors:** Atefeh Taherian Fard, Hannah C. Leeson, Julio Aguado, Giovanni Pietrogrande, Dominique Power, Cecilia Gómez-Inclán, Huiwen Zheng, Christopher B. Nelson, Farhad Soheilmoghaddam, Nick Glass, Malindrie Dharmaratne, Ebony R. Watson, Jennifer Lu, Sally Martin, Hilda A. Pickett, Justin Cooper-White, Ernst J. Wolvetang, Jessica C. Mar

**Affiliations:** 1 Australian Institute for Bioengineering and Nanotechnology, The University of Queensland, St Lucia, Australia; 2 Children’s Medical Research Institute, University of Sydney, Westmead, Sydney, NSW Australia; 3 School of Chemical Engineering, Faculty of Engineering, Architecture and Information Technology, The University of Queensland, St Lucia, Australia; 4 School of Biomedical Sciences, Faculty of Medicine, University of Queensland, St Lucia, QLD Australia

**Keywords:** Cellular senescence, Gene expression heterogeneity, ESC-derived mesenchymal stem cells, Single cell RNA-seq data

## Abstract

Following prolonged cell division, mesenchymal stem cells enter replicative senescence, a state of permanent cell cycle arrest that constrains the use of this cell type in regenerative medicine applications and that in vivo substantially contributes to organismal ageing. Multiple cellular processes such as telomere dysfunction, DNA damage and oncogene activation are implicated in promoting replicative senescence, but whether mesenchymal stem cells enter different pre-senescent and senescent states has remained unclear. To address this knowledge gap, we subjected serially passaged human ESC-derived mesenchymal stem cells (esMSCs) to single cell profiling and single cell RNA-sequencing during their progressive entry into replicative senescence. We found that esMSC transitioned through newly identified pre-senescent cell states before entering into three different senescent cell states. By deconstructing this heterogeneity and temporally ordering these pre-senescent and senescent esMSC subpopulations into developmental trajectories, we identified markers and predicted drivers of these cell states. Regulatory networks that capture connections between genes at each timepoint demonstrated a loss of connectivity, and specific genes altered their gene expression distributions as cells entered senescence. Collectively, this data reconciles previous observations that identified different senescence programs within an individual cell type and should enable the design of novel senotherapeutic regimes that can overcome in vitro MSC expansion constraints or that can perhaps slow organismal ageing.

**Supplementary Information:**

The online version contains supplementary material available at 10.1007/s11357-023-00829-y.

## Introduction

Cellular senescence is one of the main drivers of ageing and age-associated diseases [1]. This state of permanent cell cycle arrest can be induced by cell stressors like telomere attrition, oncogene activation and genotoxic or oxidative stress [2]. Senescent cells secrete a set of pro-inflammatory cytokines, chemokines, growth modulators, proteases and other soluble signalling factors that are collectively termed the senescence-associated secretory phenotype (SASP). The make-up of the SASP secretome can vary depending on the cell type and cellular context [3] and is the vehicle through which senescent cells promote inflammation and senescence in neighbouring cells, which in turn contributes to advancing the ageing process and accelerating the onset of age-related diseases [3].

Mesenchymal stem cells (MSCs) are a useful and pertinent cell model to study ageing because the degeneration of mesenchymal tissues [4] is a key component of the pathology of accelerated ageing syndromes such as progeria and Werner’s syndrome (WS) and the normal ageing process. The transplantation of healthy MSCs into a WS mouse model was shown to improve mean life span and bone density [5], underlining the importance of MSCs as a relevant cell type to study ageing and highlighting their potential in regenerative medicine applications. Because MSC are immunomodulatory and can be sourced from a range of tissues such as bone marrow or fat, they are an attractive cell type for cellular therapies [6]. However, to reach the relevant cell numbers required for clinical trials, MSC typically need to be expanded in vitro for prolonged periods of time. Similar to what occurs in vivo, this extended cell division causes many MSCs to adopt a senescent phenotype [6, 7], and this occurs at even earlier passages when primary MSCs are sourced from donors of advanced age [8]. Significantly, senescent MSCs not only enter a state of permanent cell cycle arrest but also exhibit reduced immunomodulatory properties and an increased propensity to differentiate into fat cells at the expense of differentiation into bone or cartilage [9, 10], further limiting their clinical efficacy. Studies in mice and humans have predominantly implicated telomere shortening with the onset of senescence in MSCs, a process that is modulated through the p53 and p16/pRb tumour suppressor pathways as well as GATA4-regulated pathways [11], but increased oxidative stress, mitochondrial defects, genotoxic damage, altered regulation of autophagy and changes in chromatin organisation [12] have all been implicated in promoting replicative senescence in MSCs.

Because MSCs are a heterogeneous population that consists of stem cells and progenitor compartments, it has been particularly challenging to study replicative senescence in this cell type. Furthermore, the source of the MSCs [13–17], the stage of the cell differentiation process [15, 18, 19], cultivation times, medium composition and donor age [20-23] all differentially impact these subpopulations. In addition, the markers that are typically used to identify MSCs may differ in abundance and distribution in these heterogeneous populations [7, 24]. Moreover, while senescent MSCs are typically identified based on markers such as the expression of p16, p53, p21 and senescence-associated β-galactosidase (SA-β-gal) staining, previous studies and our data presented here indicate that these markers are not expressed homogeneously across senescent MSC subpopulations [25]. To minimize the confounding impact of these experimental factors, we opted in this study to generate MSCs from Schwann’s cell precursors that were derived from a single hESC line since the epigenome and telomeres of hESCs are set to a foetal developmental stage in this cell type. Following prolonged in vitro culture of these esMSC, automated image analysis was used to quantify senescence marker expression, cell cycle progression, as well as cellular and nuclear morphology characteristics at a single cell level, revealing substantial heterogeneity and a lack of correlation between canonical senescence markers in esMSC cultures progressively entering senescence. Single cell RNA-sequencing (scRNA-seq) provided novel insights into the reasons for this heterogeneity as it revealed that esMSC undergoing replicative senescence sequentially adopts a diversity of pre-senescent and senescent states and that a small proportion appears to have arrested in the G2M part of the cell cycle. This approach further enabled the prediction of genes and molecular pathways that are potential drivers of the different pre-senescent and senescent states in human MSCs that could potentially be targets for novel senomorphic drugs.

## Materials and methods

### esMSC differentiation and culture

The hESC line Genea022 [26] was maintained under feeder-free conditions on extracellular matrix (ECM, Sigma)-coated plates with mTeSR™ Plus (Stemcell Technologies). hESC was passaged using EDTA at 70–80% confluence approximately every 5 days. For Schwann’s ell precursor (SCP) differentiation, hESCs were plated as single cells at a density of 90,000 cells/cm^2^. The next day, the culture medium was switched to SCP differentiation medium: DMEM/F12 supplemented with 1X B-27 without vitamin A, 1X GlutaMax, 1X NEAAs, 1X PenStrep, 100 µM β-mercaptoethanol, 10 µg/mL holo-transferrin, 10 ng/mL heregulin B, 3 µM CHIR, 10 µM SB431542, 50 µg/mL ascorbic acid, and 8 ng/mL bFGF. Cells were passaged every 4–5 days with Accutase and maintained in differentiation medium for 21 days. SCPs were next FACS purified with MCAM-antibodies and cultured for 3 weeks in DMEM with low glucose (1 g/L) supplemented with 10% FBS and 1X PenStrep to foster differentiation into esMSCs. At this timepoint (T0), esMSC’s identity and purity were confirmed via IHC and flowcytometric analysis of MSC marker expression (CD105 + , CD73 + , CD90 + , CD34 − , CD14 − and CD19 −) using a human MSC analysis kit (BD Biosciences, catalogue 562245) according to manufacturer guidelines and aligning with previously described international standards [27]. Subsequently, esMSCs were passaged using TrypLE at 80% confluence approximately every 5 days and plated in tissue-culture-treated flasks. The time between passages increased with increasing senescence levels. For the proliferation experiment, MSC differentiation replicates 1, 2 and 3 frozen at the different timepoints T0, T1 and T2 were revived and left to recover overnight. The next day, cells were detached, counted and plated in quintuplicate in 96-multiwell plates (PerkinElmer) at a confluence of 5000 cells/well. The following day, cells were incubated with BrdU at a final concentration of 20 µg/mL for 24 h. At the end of the incubation period, cells were washed twice with PBS and fixed with 4% PFA for 10 min at room temperature.

### Senescence-associated β-galactosidase assay (SA-β-gal)

esMSCs were washed in PBS, fixed for 10 min in 4% PFA, and incubated at 37 °C (in the absence of carbon dioxide) with fresh SA-β-gal stain solution (pH 6.0): potassium ferricyanide 5 mM, potassium ferrocyanide 5 mM, sodium dihydrogen phosphate 0.4 M, sodium hydrogen phosphate 92 mM, sodium chloride 150 mM, magnesium dichloride 2 mM and 1 mg mL^−1^ of 5-bromo-4-chloro-3-indolyl-β-D-galactopyranoside. Staining was evident in 2–4 h and maximal in 12–16 h.

### Western blotting

esMSCs were lysed with RIPA buffer containing protease and phosphatase inhibitors, and samples were prepared at 30 µg of protein with DTT (100 mM) and 1X Laemmli SDS loading dye. Lysates were resolved using denaturing TGS (Tris/glycine/SDS) buffer-based polyacrylamide gel electrophoresis (SDS-PAGE) followed by wet transfer (Tris/glycine/methanol) to nitrocellulose membranes. Primary antibodies Sox10 (rabbit, Cell Signalling Technologies [CST] #89356, 1:1000), p21 (rabbit, CST #2947, 1:1000) and B-actin (mouse, CST # 3700, 1:5000) were incubated at 4 °C overnight and HRP-conjugated secondary antibodies for 1 h at room temperature. Cross-reactivity was detected using Clarity ECL (Bio-Rad), and captured images were analysed using Image Lab 4.1 (Bio-Rad, USA) software.

### Immunochemistry

esMSCs were plated on ECM-coated 96-well imaging plates (Costar) and allowed to adhere overnight. Cells were washed once with PBS prior to 10 min of fixation with cold 4% paraformaldehyde. Cells were permeabilised with Triton-X100 at 0.1% and blocked with 3% BSA for 1 h. Primary antibodies (anti-p21 (Cell Signalling, 2946S, 1:400), anti-BrdU (Abcam, AB6326-100UG, 1:400) and anti-p16 (Abcam, AB108349-100UL, 1:400)) were incubated overnight at 4 °C. Following three PBS washes, secondary antibodies (anti-mouse IgG 488, Invitrogen, A11029, 1:400; anti-rat IgG 647, Abcam, AB150167-500UG, 1:400 or anti-rabbit IgG 568, Invitrogen, A10042, 1:400) were incubated for 45 min at room temperature. Nuclei were counterstained with Hoechst 33342 (Invitrogen, H3570, 2 µg/mL) or 4′,6-diamidino-2-phenylindole (Thermo Scientific, 62248, 1 μg/mL) prior to imaging.

Fluorescence images were acquired using an Operetta CLS high-content analysis system with a 10 × objective. All the images were analysed using the same pipeline for experimental and biological replicates with CellProfiler software. The individual cells were identified using the nuclear Hoechst counterstain. Hoechst fluorescence intensity was used to quantify genomic DNA content (‘nuclear intensity’). Other nuclear characteristics like size and circularity were also analysed. All the immunofluorescence signals within the nuclei were considered for analysis along with the extra-nuclear signal of the SA-β-gal activity that was detected in the 647 nm channel.

### Analysis for molecular marker intensities from single cell imaging

We used the interquartile range (IQR) to identify and remove outliers in the intensity data. A data point was identified as an outlier if it was above the 75th or below the 25th percentile by a factor of 1.5 times the IQR. The data was scaled by dividing each intensity value by the maximum intensity for each marker across timepoints. Wilcoxon’s ranked test (*P*-value < 0.05) was used to measure the difference between marker intensities.

### DNA damage and telomere dysfunction induced focus (TIF) analysis

esMSCs were grown on Alcian blue coated coverslips for 24 h. The following day, the coverslips were rinsed in PBS and then fixed for 10 min in freshly prepared 4% paraformaldehyde. Cell permeabilization was performed for 10 min using KCM buffer (120 mM KCl, 20 mM NaCl, 10 mM Tris–HCL pH 7.5, 0.1% Triton X-100). Coverslips were blocked with antibody-dilution buffer (20 mM Tris–HCl pH 7.5, 2% (w/v) BSA, 0.2% (v/v) fish gelatin, 150 mM NaCl, 0.1% (v/v) Triton X-100 and 0.1% (w/v) sodium azide) for 1 h at room temperature followed by incubation with a ɣH2AX antibody (05–636 Sigma-Aldrich, anti-phospho-histone H2A.X (Ser139) antibody, clone JBW301) overnight at 4 °C. The next day, 3 × 10 min PBS washes were performed; coverslips were incubated with a fluorophore-conjugated secondary antibody for 1 h at room temperature, followed by three more PBS washes. Next, coverslips were fixed again with 4% PFA for 15 min at room temperature. Cells were then dehydrated with an ice-cold ethanol series of 70%, 80% and 90% and dried and incubated with a TAMRA–OO-(CCCTAA)_3_ telomeric PNA probe (Panagene) prepared at 0.3 μg/mL in PNA hybridization solution (70% deionized formamide, 0.25% (v/v) NEN blocking reagent (PerkinElmer), 10 mM Tris–HCl, pH 7.5, 4 mM Na_2_HPO_4_, 0.5 mM citric acid and 1.25 mM MgCl_2_) for 10 min at 80 °C. Hybridization was then allowed to occur overnight at room temperature in a humidified chamber. The following day, coverslips were washed for 5 min each in 50% deionized formamide in 2X SSC, 2X SSC and 2X SSC + 0.1% Tween 20, at 43 °C. Finally, cells were counterstained with DAPI and mounted in ProLong™ gold antifade reagent. Microscopy images were acquired on a Zeiss Axio Imager microscope with appropriate filter sets. Images were analysed for telomere intensity, ɣH2AX foci and telomeres colocalising with ɣH2AX using CellProfiler v2.1.1 [28].

### Library preparation and scRNA-sequencing

Cells were harvested with TrypLE, and dead cells were stained with propidium iodide (PI). Live cell FACS was used to collect a healthy population of single cells for single cell RNA-sequencing. FACS-sorted single cell suspensions were spun down, and a cell count was performed to determine post-sort viability and cell concentration (concentration range 7.40E + 05 − 2.34E + 06, viability 85–94%). Single cell suspensions were partitioned and barcoded using the 10X Genomics Chromium Controller (10X Genomics) and the Single Cell 3′ Library and Gel Bead Kit (V2; 10X Genomics; PN-120237). The cells were loaded onto the Chromium Single Cell Chip A (10X Genomics; PN-120236) to target 10,000 cells. GEM generation and barcoding, cDNA amplification and library construction were performed according to the 10X Genomics Chromium User Guide. Reactions were performed in a C1000 touch thermal cycler with a Deep Well Reaction Module (Bio-Rad). Eleven cDNA amplification cycles were performed, and half of the cDNA was used as input for library construction. A total of 10–13 SI-PCR cycles was used depending on amount of input cDNA. The resulting single cell transcriptome libraries contained unique sample indices for each sample. The libraries were quantified on the Agilent Bioanalyzer 2100 using the High Sensitivity DNA Kit (Agilent, 5067–4626). Libraries were pooled in equimolar ratios, and the pool was quantified by qPCR using the KAPA Library Quantification Kit—Illumina/Universal (KAPA Biosystems, KK4824) in combination with the Life Technologies ViiA 7 real time PCR instrument. After the initial sequencing run, libraries were re-pooled according to estimated captured cells as determined using the Cell Ranger software (10X Genomics). Denatured libraries were loaded onto an Illumina NextSeq-500 and sequenced using a 150-cycle High-Output Kit as follows: 26 bp (Read1), 8 bp (i7 index) and 98 bp (Read2). Read1 supplies the cell barcode and UMI, i7 the sample index and Read2 the 3′ sequence of the transcript. In total, 5 sequencing runs were performed.

### scRNA-seq data analysis

#### Pre-processing and quality control

Our experimental design consisted of three timepoints with three biological replicates performed for each timepoint, resulting in nine separate samples, collectively consisting of 119,454 total sequenced cells with an average of 13,273 cells per sample. The unfiltered unique molecular identifier (UMI) count matrix from the nine libraries (three replicates for each of the three timepoints) was generated using Cell Ranger (v3.0.2). Reads were mapped to human GRCh38 genome, and an average of 92% reads was mapped to the transcriptome. True cells were identified, and droplets filtered out through DropletUtils R package. Briefly, this method computes the upper quantile of the top expected barcodes and orders them based on the library size. Any barcode containing more molecules than the 10% of the upper quantile was considered a cell and retained for further analysis. Genes with less than 10% expression across all cells were also filtered out. The scRNA-seq dataset consisted of 22,609 mean reads, 2527 median genes and 9831 median UMI counts per cell (Table S1).

#### Normalisation and integration

Seurat’s [29] integration and SCTransform pipeline were used to integrate the samples from the three replicates, and the count matrix was normalised using the default parameters. Following correction for batch effect with Seurat’s integration method and normalisation via a regularised negative binomial regression model [29], 86,771 good-quality cells were retained for the subsequent analysis. After the elimination of genes with extremely low expression (as defined as UMI counts in less than 10% of all cells), 6908 genes were retained (out of a total of 32,838 genes) (Table 1). The data was SCT normalised using 3000 highly variable features.

**Table 1:** Count matrix dimension before and after pre-processing and filtering

Count matrix	Timepoint (samples)			Replicate (samples)		
	T0	T1	T2	R1	R2	R3
Original 32,838 features × 101,945 samples	31,065	31,065	44,693	43,356	27,200	31,389
Filtered 6908 features × 86,771 samples	27,735	18,389	40,647	33,202	23,975	29,594

#### Dimensionality reduction and subgroup identification

Principal component analysis (PCA) was performed in the Seurat package. The first 20 PCs were kept based on the eigenvalues and passed into UMAP for two-dimensional visualisation using default parameters. To identity the number of clusters that best describes the heterogeneity in the single cell population, clustering at 1.2, 0.8, 0.6, 0.5, 0.4 and 0.2 resolution was applied. The result was evaluated and visualised using the clustree software package (v0.2.0). Clusters resulting from the 0.5 resolution were selected as the identity classes for cluster-specific marker identification.

#### Cell cycle assignment and differential expression analysis

Cell cycle assignment was performed according to [30], as a part of the Seurat package. To identify the differentially expressed (DE) genes specific to each timepoint and cluster, Wilcoxon’s rank sum test was used. Genes with average expression fold-change of less than − 0.25 or greater than 0.25 (with a Bonferroni’s adjusted *P*-value < 0.001) were selected for downstream functional annotation analysis and trajectory inference. Pathway over-representation analysis for each gene set was performed using clusterProfiler [31] against KEGG, Hallmark and GO Biological Process signatures. Statistical significance was defined for gene set terms with FDR corrected *P*-value < 0.05. Gene families were identified using the MSigDB database [32] and SASP atlas [33].

#### Functional enrichment analysis

The functional interaction networks were constructed by integrating protein–protein interaction (PPI) network and Spearman’s correlation of gene pairs at each timepoint. Genes/proteins were extracted from the built-in reference PPI network in R package SCENT(v1.0.2) [34] (that is obtained by integrating various interaction databases in Pathway Commons [35]), and edges were represented by Spearman’s correlation computed from the expression of genes at each timepoint. Edges with high correlation (i.e., *ρ* >|0.5|) were retained for further analysis. Network modules (densely connected regions) were identified using the MCODE app [36] in Cytoscape (v3.8.2). The same workflow was applied for cluster-specific network analysis. The power-law degree distribution was computed in Cytoscape. The regulatory interactions between gene pairs were obtained from ReactomeFIViz app in Cystoscape which is connected to the reactome pathway database [37]. Pathway over-representation analysis for each module was performed through MSigDB [32] against GO Biological Processes, the KEGG pathway database and Hallmark gene sets.

#### Pseudotime analysis and trajectory inference

Slingshot R package (v1.6.0) [38] was used to construct the pseudotime trajectory and scShapes R package (v1.0.0) [39] to identify the differentially distributed genes.

## Results

### esMSCs undergo replicative senescence in culture

Replicate cultures of esMSCs were cultured for three timepoints corresponding to 0, 26 and 63 days. At each timepoint, we used high-resolution imaging to characterise cellular features of senescence at the level of individual cells. Cells from these same timepoints were also profiled using scRNA-sequencing to characterise different states of replicative senescence.

**Fig. 1:**
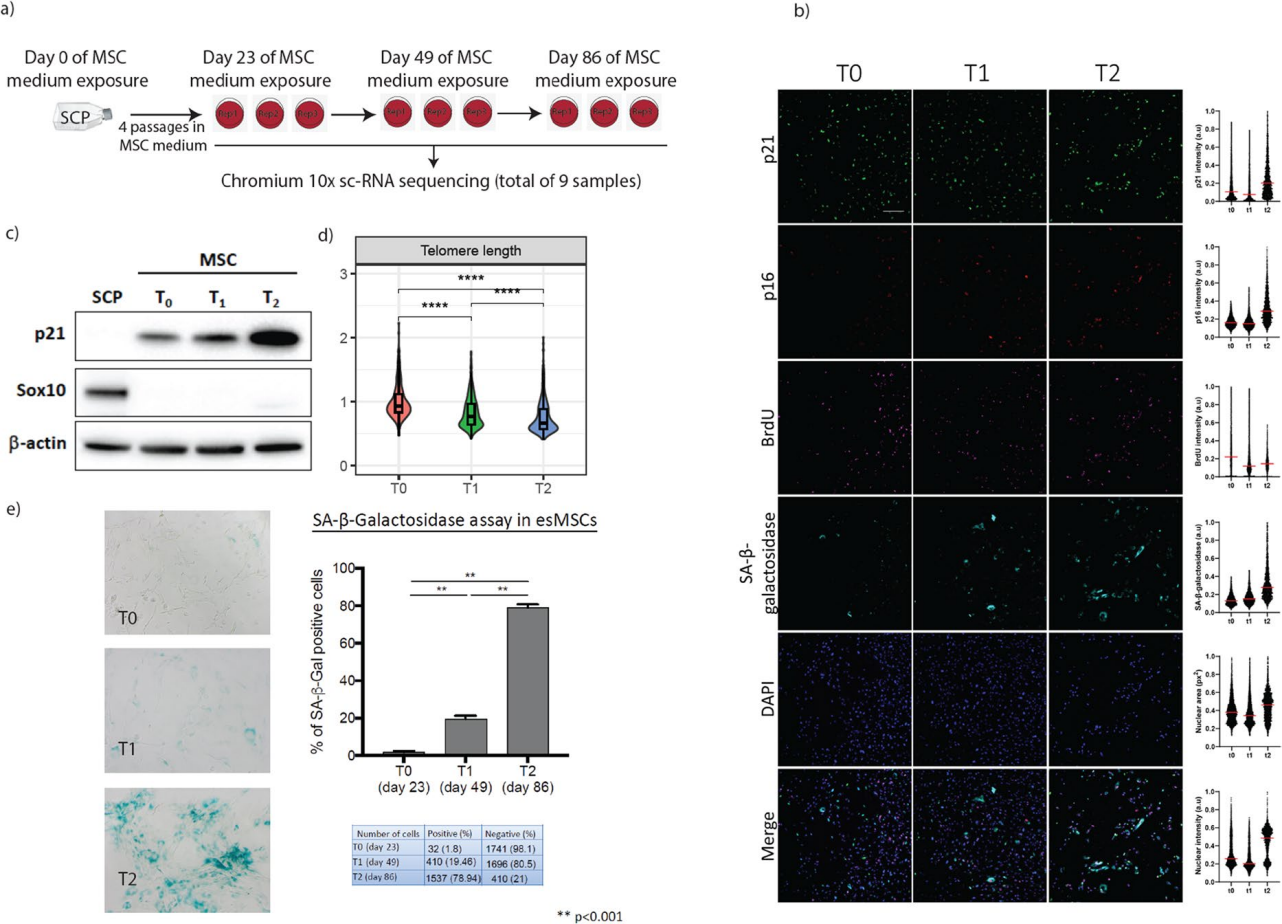
Establishing the human embryonic stem cell (hESC) derived mesenchymal stem cell (esMSC) line to study senescence. **a** Schwann’s cell precursors (SCPs) were transdifferentiated into MSCs. Young esMSCs were cultured until population doubling. Days 23, 49 and 86 post MSC medium exposure were selected as T0, T1 and T2 respectively. Cells from each of the three technical replicates at each timepoint were harvested for scRNA-sequencing. **b** Fluorescence analysis of senescence-associated markers at different timepoints. The following parameters were analysed per individual nuclei: immunofluorescence of p21 and p16, BrdU incorporation, SA-β-galactosidase activity and DAPI on MSCs cultured for different times named T0, T1 and T2 (23, 46 and 86 days, respectively). The intensities correspond to the total intensity quantified within nuclei, except for SA-β-galactosidase for which the intensity was recorded from an expansion of the nuclei, a proxy for cytoplasmic intensity. Scale bar, 100 μm. On the right side of the figure, a distribution plot profile is shown for each marker analysed. **c** Western blot analysis to confirm the conversion of SCPs to MSCs by quantifying the abundance of MSC- (p21) and SCP- (SOX10) specific markers. **d** Quantitative estimation of telomere length shows a gradual decrease in telomere length as cells enter senescence. **e** Summary of SA-b-Gal analysis along with example images and quantification. In T2, ~ 80% of the cells stained positive for this marker, indicating that most of the cells in T2 are indeed senescent

The systematic analysis of senescence in primary adipose or bone marrow-derived human mesenchymal stem cells (MSC) has been difficult because of variability in donor age, genetic make-up, tissue source and culture medium. To minimize these confounding factors, we examined replicative senescence in MSC derived from the healthy control pluripotent stem cell line GENEA 22, since ageing-associated epigenome changes and telomere length exhibit a foetal developmental make-up in this cell type. To generate defined populations of esMSC, we first subjected this hESC line to a differentiation protocol that generates cranial Schwann’s cell precursors (SCPs) that express SOX10 and MCAM (3 independent differentiations). Culturing MCAM-sorted SCPs from each replicate in FCS-containing MSC culture medium for a 3-week period fostered their homogeneous transition into cranial esMSC (Fig. 1a). At this timepoint (which we designated timepoint zero T0), esMSCs exhibited the spindle-like morphology and surface marker expression profile of primary MSCs (CD105 + , CD73 + , CD90 + , CD34 − , CD14 − and CD19 − ; Figs. 1b and S1). To study replicative senescence in these esMSCs, we cultured newly generated esMSC (T0) for 26 (T1) and 63 (T2) days (Fig. 1a, b). We observed that over this time period, the cell shape of the esMSC became increasingly irregular and larger (Fig. 1b) until at day 63 (T2, passage 12 for replicates 2 and 3) most cells had adopted the large and flattened appearance characteristic of senescent MSCs (Fig. 1b). In agreement with this notion, the esMSC cultures exhibited increasing proportions of cells that showed senescence-associated β-gal enzyme activity (SA-β-gal) over time (T0: 1.8%, T1: 19.5% and T2: 78.9%) (Figs. 1e and S2). This progressive increase in SA-β-gal expressing esMSC was accompanied by a decrease in telomere length over time (Fig. 1d and Figs. S2 and S3) and a concomitant decrease in BrdU incorporation, indicating a progressively lower proliferation rate of these cells over time in culture (Fig. S2). Automated image analysis of > 15,000 cells from replicate MSC cultures at each timepoint revealed a progressive temporal increase and shifts in population distribution of the canonical senescence markers SA-β-gal, p16 and p21 (Figs. 1b and S2). In agreement with this data, western blot analysis of protein lysates from the esMSC cultures revealed a robust increase in the CDK (cell cycle) inhibitor p21 (CDKN1A) with increased passage number and confirmed the absence of the Schwann cell precursor marker SOX10 (Fig. 1c). SenezRed, another marker related to senescence, did not change significantly across timepoints (Fig. S4), which aligns with a previous study that found evidence of telomere erosion but no mitochondrial changes in long-term cultured primary human bone marrow MSCs [40]. The number of γH2AX stained cells and telomere damage induced foci (TIF), both hallmarks of senescence [41], indicated that esMSCs accumulate DNA damage in the form of double strand breaks over time in culture, although the changes in numbers of TIF were not statistically significant (one-way ANOVA, *P*-value > 0.01) (Fig. S3). Importantly, the expression of MSC marker expression did not significantly change between T0, T1 or T2, and esMSCs from each timepoint continued to meet the standard requirements for MSC characterisation and conformed to the Rohart test gene signature of MSC identity (Fig. S5) [39].

#### esMSCs undergoing replicative senescence display variable senescence marker expression profiles at a single cell level

**Fig. 2:**
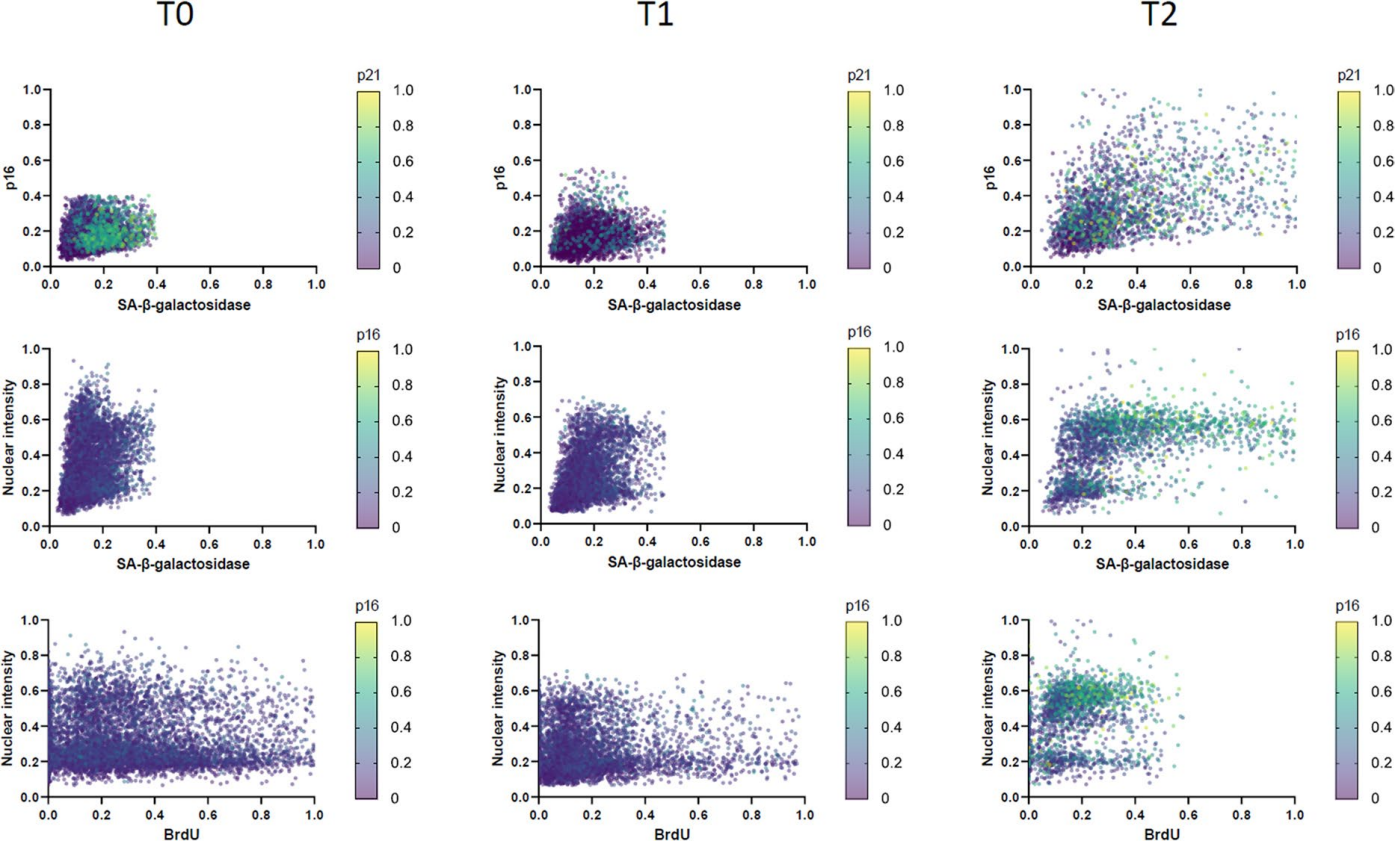
Correlation plot of nuclear p16 and p21, DNA content, SA-β-galactosidase activity and BrdU across T0, T1 and T2. Each dot represents a single cell (n > 2400)

Our automated image analysis platform provided an opportunity to quantify senescence markers at the single cell level, and we next investigated how consistent canonical markers of senescence (SA-β-gal, p21, p16 and loss of BrdU incorporation) co-occurred at the single cell level. This revealed that, as expected, BrdU incorporation was inversely correlated with increased SA-β-gal and p16 expression for the vast majority of cells over the entire time course (Figs. 2 and S6a–b). However, p21 expression already increased in many esMSC at the intermediate T1 timepoint in the absence of increases in these senescence markers. Interrogation at the single cell level also revealed the emergence of two cell subpopulations with differing DNA content that were both positive for SA-β-gal activity and p16 expression, and displayed low BrdU incorporation, suggesting either senescence of esMSC in the G2M phase and/or aneuploidy of senescent cells.

Collectively, these data indicated that while canonical senescence markers demonstrably shift at a population level, there is a considerable heterogeneity between these markers at a single cell level, suggestive of the presence of multiple senescence programs and/or perhaps different or transitional senescent cell populations within the cultures at different timepoints.

### Single cell RNA-sequencing reveals the identity and temporal relationships of pre-senescent and senescent cell states in human esMSC cultures undergoing replicative senescence

**Fig. 3:**
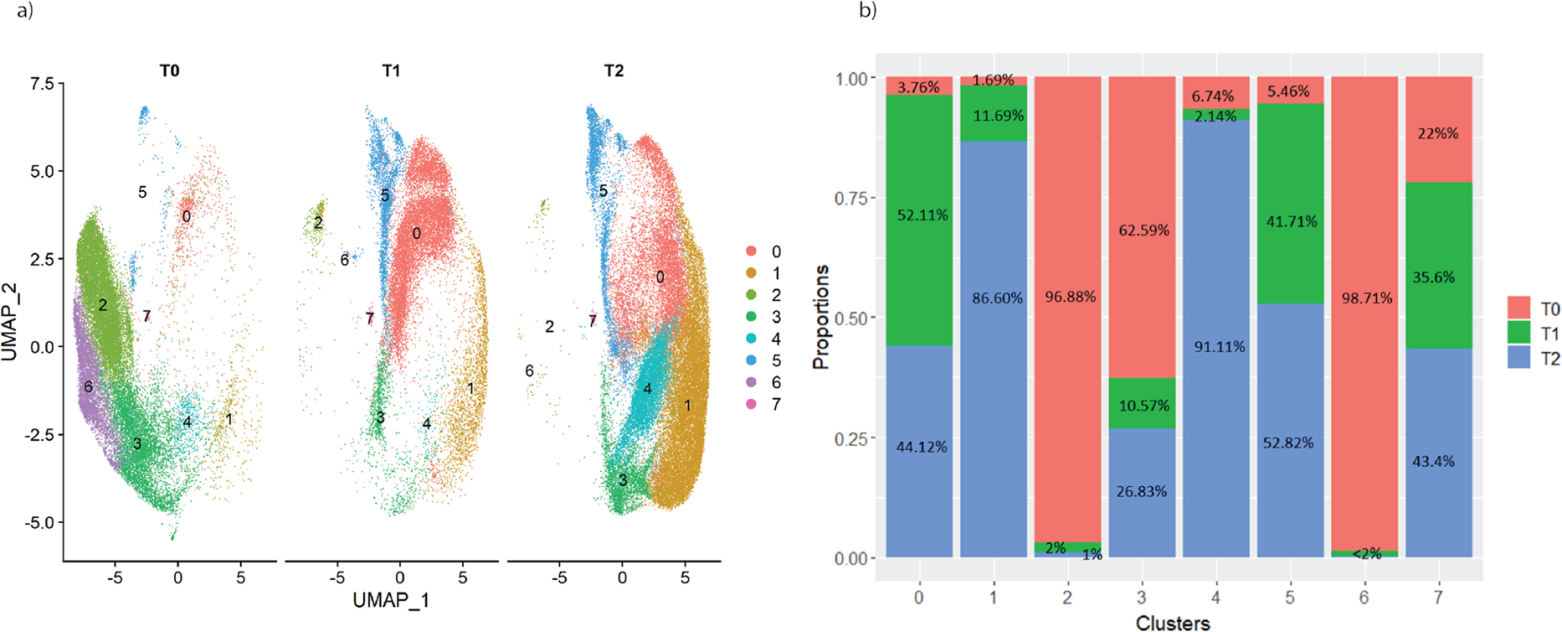
The overall representation of the esMSC population at different timepoints. **a** UMAP of esMSC colour coded by clusters, split by timepoint. **b** Proportion of cells in each cluster with respect to timepoints. Clusters 2 and 6 are mainly comprised of T0 cells, whereas the majority of cells in clusters 1 and 4 are from T2 (senescent) cells. Clusters 0 and 5 comprised of mixture of T0, T1 and T2 cells

To gain further insight into the intercellular heterogeneity of esMSC undergoing replicative senescence, we next performed single cell RNA-sequencing of replicate cultures harvested at T0 (freshly generated eMSC), T1 (cultured for 26 days) and T2 (cultured for 72 days), capturing transcriptomic profiles for 86,771 high-quality cells. UMAP comparisons revealed excellent overlap of the three biological replicates (each consisting of pooled triplicate samples) (Fig. S7). Furthermore, cells from each timepoint predominantly clustered together, suggesting a largely synchronous transition of cells into distinct transcriptional states as the esMSC underwent replicative senescence (Fig. S8). Clustering revealed that cells at T0 predominantly consist of clusters 2, 3 and 6. T1 cells consist predominantly of cluster 0 cells, cluster 5 cells and a minor cluster 7, while T2 cells mainly consist of clusters 1 and 4 and subsets of cluster 0 and cluster 3 cells (Fig. 3).

To examine the contribution of cell cycle effects to this differential clustering, well-annotated genes were used to assign cell cycle phases to each cluster, revealing that cells in T0 were predominantly in G1-, S- and G2M-phases, whereas cells in T1 mainly consisted of G1-phase cells with a minor proportion of cells in S-phase. Cells from T2 were represented by G1-phase cells, which was consistent with the reduced BrdU incorporation into esMSCs at this timepoint but also contained G2M-phase cells that mapped to cluster 3 (Figs. S7a–b and S8).

**Fig. 4:**
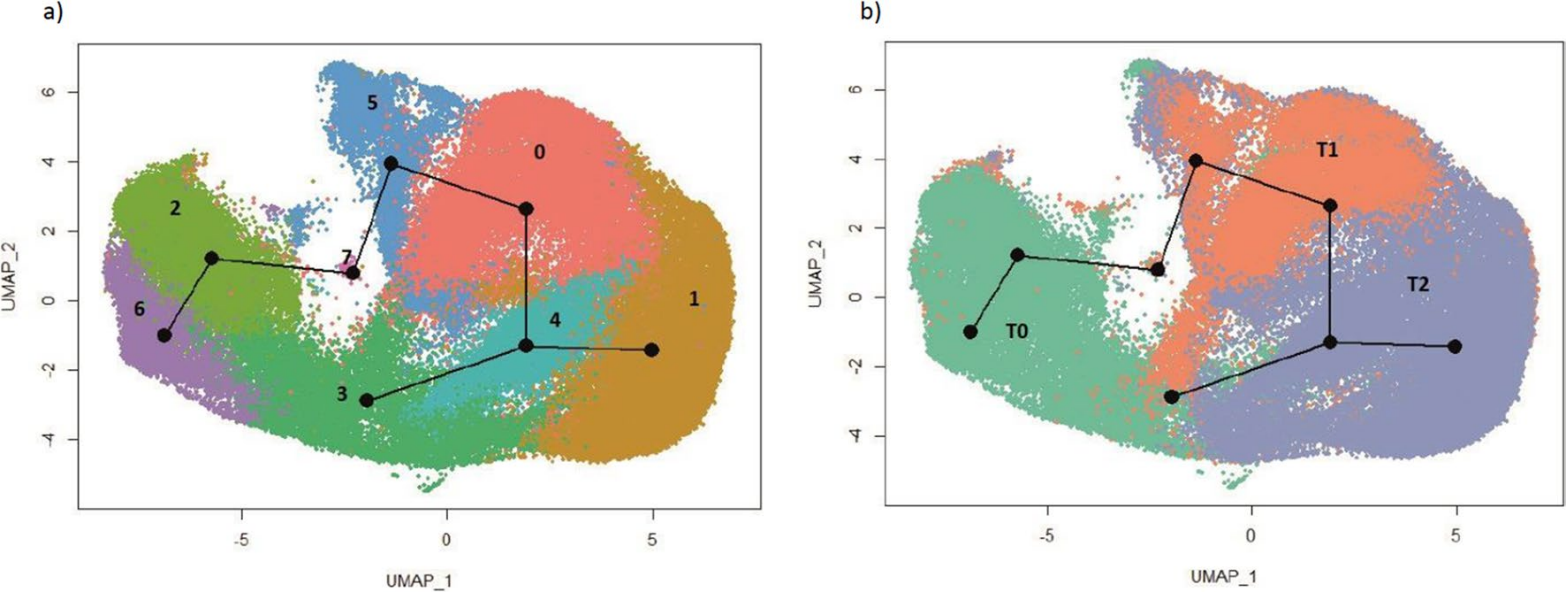
Trajectory inference analysis. The UMAP is colour coded based on **a** cluster and **b** timepoints. The trajectory starts at cluster 6 and ends at two different senescence states: oncogene-related senesce and T2-G2M cells (cells that were permanently arrested in G2M)

To predict the transitions that occur between cell clusters, we next used pseudotime trajectory mapping of the scRNA-seq data. This analysis revealed that esMSC clusters 2 and 6 from T0 transition to cluster 5 and then into cluster 0 cells at T1 via a small group of transitional cells (cluster 7). Cluster 0 cells are next predicted to transition into cluster 4 which next splits into two different cell states, either cluster 1 or a subset of cluster 3 cells (Fig. 4).

The trajectory map revealed transitional cell states of esMSC undergoing replicative senescence that have not been previously identified. To gain insight into the molecular and cellular processes underlying esMSC replicative senescence, we examined what pathways and gene sets were enriched in each of the cell clusters and identified markers for these. Cluster 6, the start of the esMSC senescence trajectory, is distinctly enriched for pathways associated with insulin-like growth factor (IGF) receptor signalling IGFBP2 and IGFBP4 that regulate cell metabolism, development and growth (Fig. S9a). Cluster 2 cells showed a significant enrichment for pathways associated with regulation of cell shape and epithelial to mesenchymal transition (EMT) (Tables S2 and S3). Cluster 5, the next subpopulation in the temporal analysis, which consists of 41% cells belonging to T1 and 52% to T2, was enriched for genes related to extracellular matrix organisation, antigen processing and presentation of MHC Class 1 (Table S2, Fig. S9a). We hypothesize that cells in this cluster may be involved in changing the immune-modulatory properties of esMSCs. These cluster 5 cells are next predicted to transition into cluster 0 that is enriched for apoptosis and p53 pathway genes and consists of 52% T0 and 44% T2 cells. Cluster 0 cells displayed high expression of NUPR1 (Table 2), a gene that is activated under oxidative stress or ER stress conditions and is a key regulator of ferroptosis (Fig. S9b). Cluster 0 cells also highly express TRIB3, an inhibitor of cell proliferation (Fig. S9b) that becomes upregulated in response to several forms of cellular stress [54] including oxidative ER stress and hypoxic stress (pathways that are also significantly enriched in cluster 5). Of note, cluster 0 cells also strongly express ZFAS1, a long non-coding RNA (lncRNA) that promotes adipogenic vs osteogenic differentiation in MSCs, a feature associated with ageing MSCs [40].

**Table 2:** Top 5 timepoint and cluster-specific markers

	Group	No. of cells	No. of markers (up/down)	Top 5
	T0	27,735 (32%)	317 (187/130)	KRT18, IGFBP2, SHISA2, MEST, IGFBP4
	T1	18,389 (21%)	126 (77/49)	ACTA2, CRYAB, GDF15, LUM, BGN
	T2	40,647 (46%)	240 (83/157)	CCND1, SERPINE2, MMP1, MMP2, NEAT1
Early stage of time course (proliferative)	Cluster 2	13,822 (16%)	199 (105/94)	KRT18, IGFBP2, FHL1, MAP3K7CL, NREP
	Cluster 3	11,345 (13%)	360 (267/93)	HIST1H4C, UBE2S, PTTG1, H2AFZ, PCLAF
	Cluster 6	5229 (6%)	263 (125/138)	IGFBP2, IGFBP4, IGFBP3, SHISA2, CDH6
Late stage of time course (pre-senescence & senescence)	Cluster 0	22,087 (25%)	106 (31/75)	NUPR1, BGN, TRIB3, EIF3E, ZFAS1
	Cluster 1	19,851 (23%)	200 (60/140)	IGFBP5, MT2A, CCND1, SFRP1, NEAT1
	Cluster 4	7595 (8%)	149 (43/106)	S100A6, G0S2, PCSK1N, CYP1B1, GNG11
	Cluster 5	6683 (7%)	659 (491/168)	POSTN, HSPA5, UBC, HSP90AA1, ACTA2
	Cluster 7	159 (< 1%)	940 (137/778)	MALAT1, NEAT1, S100A11, H3F3B, UBA52

**Fig. 5:**
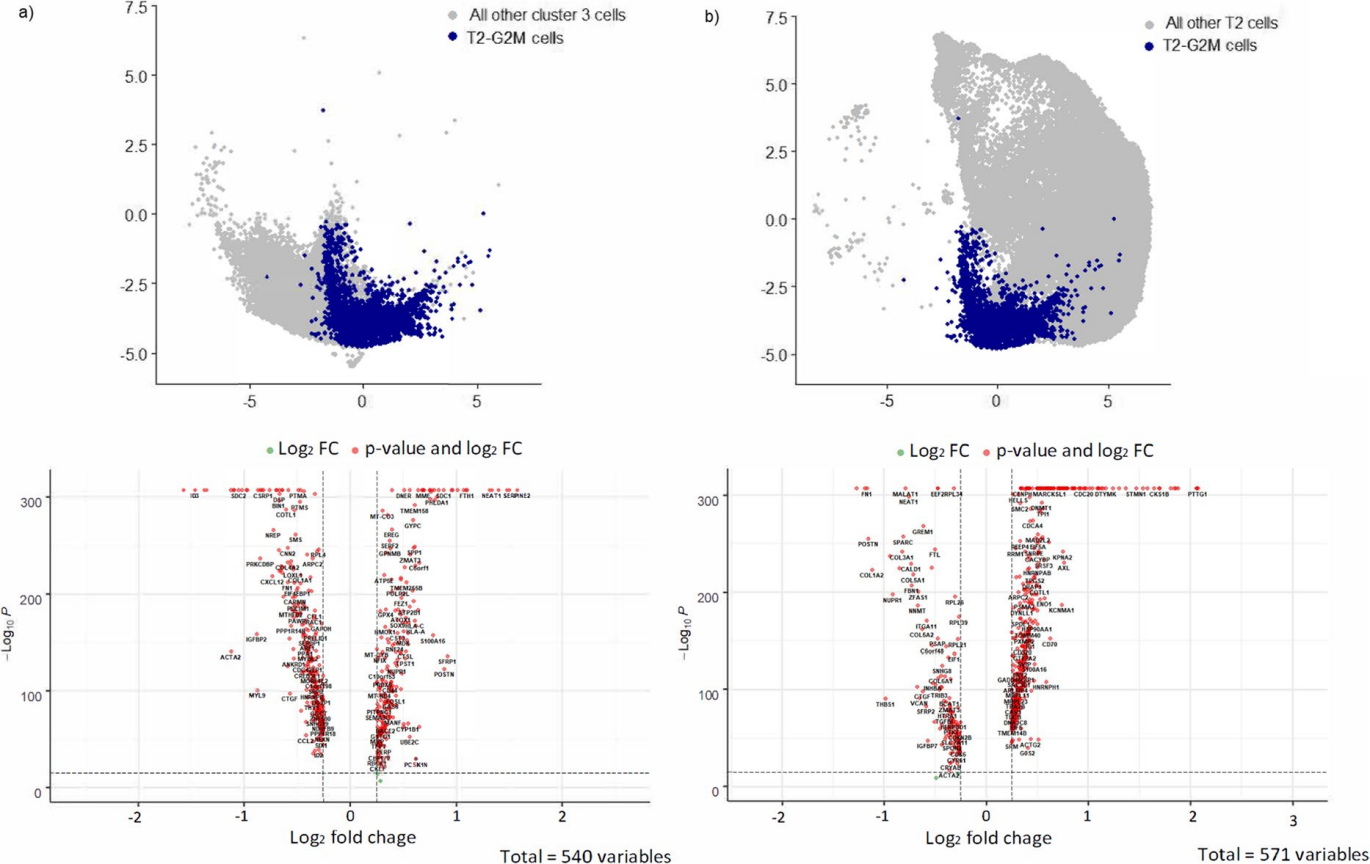
Differential expression analysis of T2-G2M cells (cells that were permanently arrested in G2M, in blue) with **a** proliferative esMSC subtype-3 (other cells in cluster 3) and **b** other senescent cells (other T2 cells). Top panel: UMAP highlight cells in the comparison. Bottom panel: significant DE genes (LogFC × >|0.25|, Bonferroni’s adjusted *P *- value < 0.001)

Cluster 0 cells are predicted to next transition into cluster 4 which is enriched for oncogenes or tumour suppressor genes (23.48%), suggesting these correspond to cells undergoing oncogene-associated senescence (Fig. S10, Table S3). Standout upregulated genes include G0-G1 switch gene 2 (G0S2) (Fig. S9b) a tumour suppressor gene associated with human dermal fibroblasts senescence, haematopoietic stem cell quiescence, adipocyte differentiation and cell-cycle withdrawal [6]. Another notable gene in cluster 4 is transcription elongation factor A protein-like 7 (TCEAL7), a gene known to regulate human telomerase reverse transcriptase (hTERT) expression and telomerase activity by inhibiting the c-Myc pro-oncogene in cells that have activated the alternative lengthening of telomeres (ALT) mechanism. This is significant as more than 70% of mesenchymal tumours use the ALT pathway to maintain telomere length and bypass replicative senescence [7, 8]. Cluster 4 cells are predicted to either transition into cluster 1 cells that have enrichment for TGF-β signalling and markers for this cluster include pro-inflammatory SASP genes such as growth differentiation factor 15 (GDF15) [9], THBS1 [10], CCL2, CD70, CDKN1A, DCBLD2, EREG, HIF1A and MMP14 (Fig. S10, Table S3) or into a subset of cluster 3 cells. This subset of T2 cluster 3 cells is enriched for SASP factors (MMP1, SERPINE2 and MMP2) as compared to the cluster 3 T0 subpopulation (Fig. 5a and Table S4) and displays strong expression of CCND1, a well-established regulator of CDK kinases throughout the cell cycle, and a protein that specifically interacts and regulates CDK4/CDK6 that are required for cell cycle G1/S transition [19]. Compared to the rest of T2 cells in the dataset (Fig. 5b, Fig. S12 and Table S4), T2 subcluster 3 displays increased expression of BIRC5, an antiapoptotic gene linked to G2M cell cycle phase [20]. The fact that this cluster also uniquely over-expresses TPX2, a gene that promotes chromosomal instability [21, 22], further reinforces the notion that T2 subcluster 3 cells represent putatively oncogenic esMSC that exhibit aneuploidy. Given that this cluster also highly expresses CCNB1 (Fig. S11), a gene that has been implicated in p53-mediated permanent cell cycle arrest during senescence [41], makes it highly likely that these cells represent the SA-β-gal^high^, p16^high^ and BrdU^low^ cells with increased DNA content that we identified in our single cell profiling.

While the interpretation of these clusters largely rests on existing pathways and the current functional knowledge that links them back to cellular senescence, a major outcome of this study is also the identification of the cluster states, the trajectories that connect them and the marker genes that define them. To characterise each cluster, we also highlight the significant marker genes (top 3 based on fold change, see Fig. S9b), the top 3 Gene Ontology Biological Processes (Fig. S9a) and the top 3 cell surface receptors [42] that were marker genes for each cluster (Fig. S9c). It is likely that these cell surface receptors may serve as a useful resource for future studies that look to identify or validate these clusters in other cellular systems.

Collectively, these data indicated that healthy proliferating esMSCs undergoing replicative senescence sequentially transition into cells experiencing metabolic and oxidative stress, then into a pre-senescent state characterised by ER-stress and p53 regulated senescence, before transitioning into cells experiencing oncogene-induced senescence that split either into deep senescent cells with strong SASP secretions or into aneuploid cells that have been permanently arrested in G2M.

### Gene regulatory networks are progressively less connected as esMSCs undergo replicative senescence

Network models were constructed for each timepoint using co-expression of gene–gene pairs where pairs were retained for an existing protein–protein interaction network (using SCENT, see Methods) with high correlation (using Spearman’s |*ρ*|> 0.5). Subnetworks were identified using MCODE (see Methods). For all timepoints, the number of positively correlated edges was significantly higher than the number of negatively correlated edges, and the T1 network had the largest number of positively correlated edges compared to the T0 and T2 networks (Table 3).

**Table 3:** PPI network comparison during senescence

Subnetwork	Nodes	Edges					
		T0		T1		T2	
		Positive	Negative	Positive	Negative	Positive	Negative
1	31	105	0	450	0	94	0
2	18	28	15	10	4	5	0
3	29	29	7	41	0	26	4
4	8	19	0	0	0	0	0
5	8	3	0	4	0	9	5
6	12	6	2	8	2	5	4
7	5	7	0	1	0	0	0
8	3	2	0	2	0	1	0
Total	114	198	24	515	7	140	13

^*^Nodes are extracted from the reference PPI network via SCENT packages, and edges represent Spearman’s *ρ* >|0.5|. Subnetworks are identified with MCODE app in Cytoscape

Two subnetworks, modules 4 and 7, showed striking changes in the number of edges in the transition from T0 to T2 where edges with positive correlation were observed at T0, and this correlation was subsequently lost at T1 and T2. These genes are mainly associated with cell division and the regulation of cell cycle transition (*CDC20*, *TPX2*, *CENPF*, *UBE2*, *PPTG1*, *BIRC5* and *CKS1B*). Overall, T2 has the lowest number of total positive edges as compared to T0 and T1, and the lowest number of edges in the majority of the modules (i.e., 1, 2, 3, 4, 6, 7 and 8) (Tables 3 and S5, Fig. S13). Collectively, this data indicated that gene regulatory networks are progressively less connected as esMSCs undergo replicative senescence.

### Cluster-specific regulatory networks show a decreased degree of connectivity as esMSCs transition into senescence.

To further examine transcriptional regulation changes across the pseudotime trajectory, we next constructed cluster-specific co-expression networks. The nodes in the networks represent cluster-specific marker genes, and the edges reflect the Spearman correlation between the expression profiles of two genes. Following retention of edges with the highest correlations (i.e., *ρ* >|0.5|), we found that the final networks exhibited a power-law degree distribution with a few hub genes (Tables 4 and S7). These networks are scale-free, which means the connectivity of the network is dominated by a small number of hub genes which are the most highly connected in the network and surrounded by a larger group of weakly connected genes. Highly connected genes in clusters 6 and 2 (T0) (Fig. S13) are associated with cell proliferation (e.g., *IGFBP5*, *TPM1*, *CNN1* and *HBEGF*). The Cluster 5-based network had the highest number of nodes and edges and was composed of 11 modules. Mitochondrial genes were represented among the top 10 highly connected nodes for the cluster 5 subnetworks, and these genes were involved in oxidative phosphorylation and the ATP metabolic process (*MT-ATP6*, *MT-CO2*, *MT-CO1*, *MT-ND4*, *MT-ND3* and *MT-CYB*). These observations are consistent with our previous trajectory inference, indicating that oxidative stress initiates a DNA damage response that leads to activation of p53 and p16 (*CDKN2A*) pathways that in turn initiate and sustain cell cycle arrest [43]. The latter also supports our hypothesis that cluster 5 cells are experiencing metabolic stress.

**Table 4:** Characteristics of cluster-specific co-expression networks

Cluster	No. of nodes	No. of edges	No. of modules	Top 10 highly connected genes
6	115	776	4	TKT, UGCG, SLC25A5, AKAP12, EEF1A1, C12orf75, NPM1, HNRNPA1, HBEGF, IGFBP5
2	84	546	3	ARPC2, CNN1, PRKCDBP, TPM1, EEF1A1, HMGCS1, IGFBP2, MAP2K2, SELENOW, COL3A1
5	396	11932	11	MT-ATP6, VCAN, MT-CO2, MT-CO1, GSTP1, MT-ND4, MT-ND3, FBN1, COL1A1, MT-CYB
0	22	48	1	TIMP1, COL1A1, RPL21, C6orf48, NPC2, VCAN, TKT, RPS3A, ZFAS1, GDF15
1	42	178	4	DCBLD2, S100A16, FTH1, SFRP1, HMGA1, THBS1, CCND1, IGFBP5, MT2A, HMGA2
4	25	92	2	S100A13, HLA-A, KCNMA1, CD63, PCSK1N, RPS27, B2M, HLA-B, HMGA1, FTL
3	260	3552	6	KNSTRN, CENPF, CCNB1, CKS2, ARL6IP1, TPX2, KPNA2, CKS1B, CDC20, BIRC5

Cells in cluster 0 express genes involved in regulating the recovery from metabolic stress such as *TIMP1* and *GDF15* (top 10 most highly connected genes in cluster 0). TIMP1 is an inhibitor of matrix metalloproteinases (MMPs) and has anti-apoptotic functions [44]. Expression of MMPs is associated with the production of reactive oxygen species (ROS) that drive the emergence of senescence and age-associated disease [45]. GDF15 is involved in the stress response program of cells following cellular injury, and its increased gene expression is associated with age-associated states such as tissue hypoxia, inflammation and oxidative stress [46]. Another gene of interest from the top 10 highly connected genes in cluster 1 (the senescence state, T2) is *SFRP1*, a gene that is commonly over-expressed in senescent cells exposed to DNA damage or oxidative stress [47].

Analysis of the cluster 4 co-expression network in the senescent esMSCs indicates that *S100A13* is the gene with the highest number of interactions. This is significant because a previous study showed that overexpression of S100A13 increased NF-κB activity and induced multiple SASP genes, resulting in the emergence of cellular senescence [48].

### Replicative senescence of esMSCs displays dynamic changes in distributions of gene expression in long non-coding RNAs and genes involved in DNA damage response and apoptosis

Capturing gene expression at the single cell level can provide information that goes beyond confirming whether a gene is differentially expressed but instead the opportunity to model any changes in the distribution of a gene’s expression profile. This type of distribution-centric analysis has demonstrated value for overcoming some of the challenges of modelling scRNA-seq data [39] like excess zeros and heterogeneous data. We investigated which genes changed their distribution between all timepoints where the selected options for distributions were one of the following, Poisson (P), zero-inflated Poisson (ZIP), negative binomial (NB) and zero-inflated negative binomial (ZINB). Generalised linear models were used to represent each of the four distributions and adjusted for variation due to biological replicates. Following the analysis framework of [39], out of the 3563 genes that pass the Kolmogorov–Smirnov (KS) goodness of fit test, 585 genes (16.4%) changed distribution in at least two timepoints (Tables 5, 6 and S6). Among these, 117 genes (20%) switched distributions at all three timepoints, that is, from T0 to T1 and from T1 to T2. These 117 genes were significantly enriched for pathways in the response to DNA damage stimuli and non-coding RNA processing pathways (Table S2). These data are in accordance with previous studies that showed lncRNAs targeting p21/p53 and pRB/p16 pathways [49] are involved in telomere length attrition [50], consistent with our data showing the shortening of telomere length in senescent esMSCs (Fig. S3).

**Table 5:** Set of genes belonging to the same family of distributions. Total of 3563 genes passed the Kolmogorov–Smirnov test

	T0	T1	T2
Poison	1568 (44%)	1742 (48.89%)	1556 (43.67%)
NB	1833 (51.44%)	1647 (46.22%)	1881 (52.79%)
ZIP	135 (3.78%)	150 (4.2%)	106 (2.97%)
ZINB	26 (0.72%)	23 (0.64%)	19 (0.53)

**Table 6:** Differentially distributed genes across timepoints

	Count	T0	T1	T2
Changing in all three timepoints Total = 117	14	Poison	NB	ZIP
	22	Poison	ZIP	NB
	1	Poison	ZINB	NB
	22	NB	Poison	ZIP
	14	NB	ZIP	Poison
	2	NB	ZINB	ZIP
	27	ZIP	Poison	NB
	15	ZIP	NB	Poison
Changing in two timepoints Total: 468	138	Poison	NB	Poison
	30	Poison	ZIP	Poison
	212	NB	Poison	NB
	69	NB	ZIP	NB
	15	NB	ZINB	NB
	1	ZIP	Poison	ZIP
	3	ZINB	NB	ZINB
	Total 585			

The link between genes that change their expression distribution shape and their role as non-coding RNAs like microRNAs and lncRNAs may represent a functional contribution towards senescence and ageing. Recently, the role that microRNAs play in regulating the stability of mRNAs after transcription has been shown to be associated with ageing [51]. Moreover, the expression of splicing factors has been linked to increasing age, and one study points to information from the expression levels of mRNA processing as a stronger indicator of age compared to transcriptional noise and the chronological age of the individual [52]. Analysis of distributional shapes of gene expression from scRNA-seq may represent a way to link subtle changes in these kinds of genes with the increasing prevalence of senescent cells in T0 to T2.

## Discussion

Replicative senescence is brought about by critically shortened telomeres that initiate DNA damage response pathways that result in permanent cell cycle arrest. Replicative senescence is an inter-cellularly heterogeneous and asynchronous process not only because telomere length and shortening rates can vary within a population of cells but also because inter-cellular variations in mitochondrial ROS production, metabolism, non-telomeric DNA damage levels, proteostasis and oncogenes can all modulate the type and severity of the telomere shortening induced DNA damage response. Furthermore, pro-inflammatory molecules of the SASP pathway that are released by cells entering replicative senescence induce secondary senescence in neighbouring cells. It is therefore not surprising that imposition of telomere shortening (e.g., by knocking out telomerase), oxidative stress, enforced oncogene expression, ER-stress, metabolic processes and production of SASP factors have all been reported to occur during replicative senescence and accelerate the process. Since to date replicative senescence has been mainly studied at the bulk-population level, it has remained largely unclear whether these processes co-occur within cells undergoing replicative senescence or are present in subpopulations of cells that temporally transition into each other. In this study, we therefore examined gene expression and senescence markers at the single cell level in human ES-cell-derived mesenchymal stromal cells (esMSC) that progressively transition into replicative senescence. Our data reveal that healthy proliferative esMSCs sequentially transition into cells that show gene expression signatures of cells experiencing metabolic and oxidative stress, then into a pre-senescent state characterised by genes involved in ER stress and p53-regulated senescence, before transitioning into cells experiencing oncogene-induced senescence. This population next splits into either deep senescent cells with strong SASP secretions or into cells with SASP and oncogene signatures that are predominantly arrested in G2M. We hypothesize that this senescent G2M population [41, 53] has acquired p53 mutations that retain nuclear CyclinB, as previously observed by others. These data also explain why we and others observe poor correlations between senescence markers such as p21, p16 and SA-β-gal and loss of BrdU incorporation at a single cell level, even though these markers significantly change at a population level. More importantly, our data for the first time temporally orders transcriptionally distinct pre-senescent and senescent subpopulations in a logical order that provides insights into the chronological progression of the replicative senescence program. Our data further show that this stepwise progressive transition into replicative senescence of esMSC is accompanied by a loss of gene regulatory network control, as was previously observed in cultured fibroblasts. As our data indicate that multiple pre-senescent and senescent states can co-occur within a senescent cell population, it is clear that senolytics that target specific pathways can only be partially efficacious in eliminating senescent cells. In addition to examining gene expression levels in individual cells, we also investigated changes in gene expression distribution, revealing that a subset of genes show conspicuous changes in gene expression distribution as cells enter senescence. We speculate that this is likely a function of altered miRNA and lncRNA expression, changes in alternative splicing of these transcripts, or changes in transcriptional bursting rates. While our experimental approach of studying replicative senescence in esMSC was designed to minimize potential confounding factors such as origin, chronological age and genetic background, we cannot exclude the possibility that selective clonal expansion during MSC generation or subsequent subculture contributes to some extent to the observed transcriptionally distinct pre-senescent and senescent cell clusters, nor can we exclude the possibility that differences in droplet capture efficiencies between senescent cells are a source of variability. Spatial transcriptomic analysis combined with lineage tracing of bar-coded replicative senescent cultures that leverage the data and cluster marker resources provided in this study should clarify these issues going forward. Despite these limitations, our data already enable the prediction of cell surface proteins that mark pre-senescent and different senescent subpopulations and provide a deeper understanding of the chronologically ordered molecular processes that govern the transition from one subpopulation into the next. Going forward, these data may enable the design of novel senomorphics that can overcome current in vitro cell expansion constraints that limit clinical trials with MSCs or that can promote healthy ageing outcomes in vivo.

### Supplementary Information

Below is the link to the electronic supplementary material.

Supplementary file1 (XLSX 11 KB)

Supplementary file2 (XLSX 284 KB)

Supplementary file3 (XLSX 223 KB)

Supplementary file4 (XLSX 136 KB)

Supplementary file5 (XLSX 31 KB)

Supplementary file6 (XLSX 25 KB)

Supplementary file7 (XLSX 10 KB)

Supplementary file8 (PDF 41922 KB)

## Data Availability

The full datasets, including raw and processed single cell RNA-seq data, are available on Gene Expression Omnibus database under accession number GSE200157.
