# Supplementary material for: Deconstructing heterogeneity of replicative senescence in human mesenchymal stem cells at single cell resolution
Source: GeroScience. 2023 Jun 14;46(1):999–1015. doi: 10.1007/s11357-023-00829-y (PMC10828319; doi:10.1007/s11357-023-00829-y)
Supplement: Supplementary file 8 — Supplementary file8 (PDF 41922 KB) [file 11357_2023_829_MOESM8_ESM.pdf]

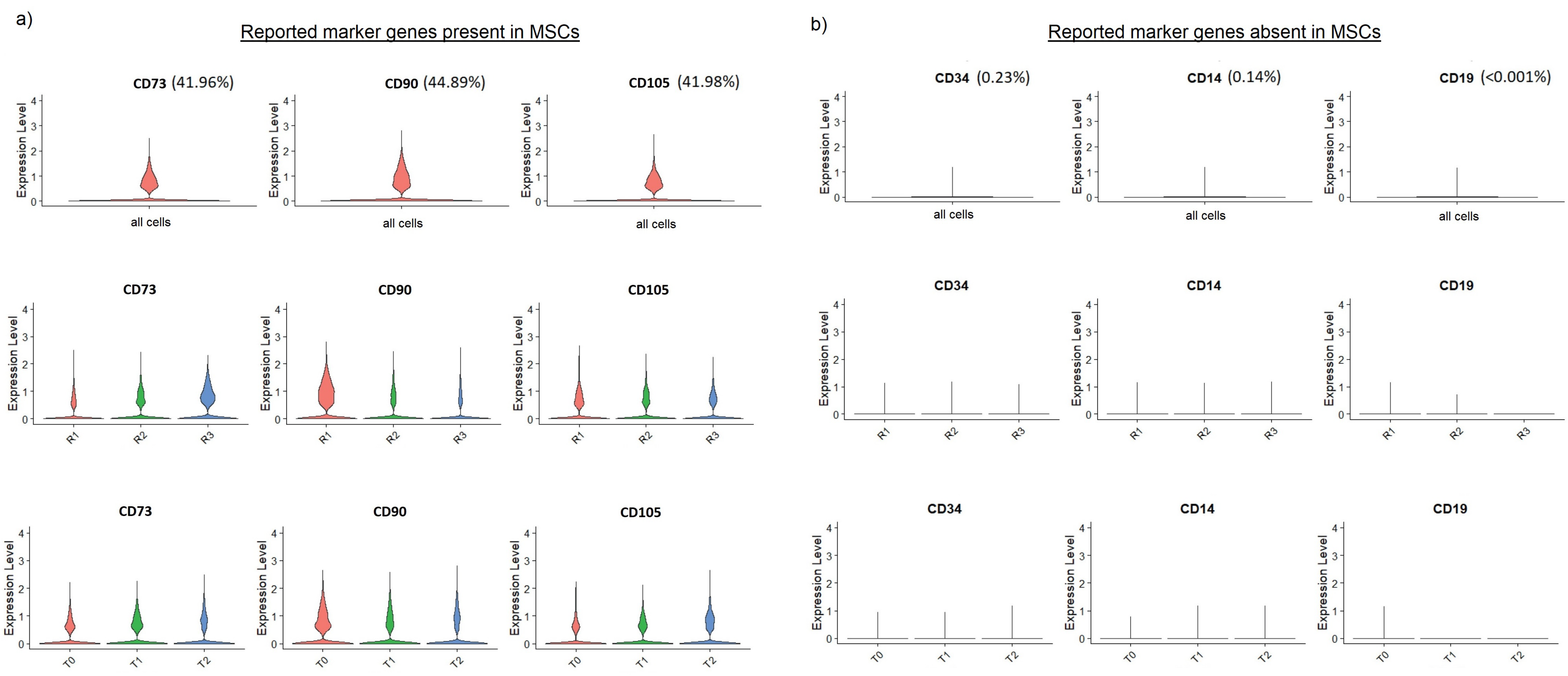

Figure S1. Expression of MSC-specific markers in the sc RNA-seq data prior to pre-processing. We inspected the scRNA-seq data to assess whether it met standard requirements for MSC characterisation. Traditionally, cells must express surface markers such as CD105 (ENG), CD73 (NT5E), and CD90 (THY1) and lack expression of several genes including CD14, CD19 and CD34. We observed that collectively, more that 79% of cells express (count > 0) either of the positive markers, and more than 99% of cells lack expression of (UMI = 0) negative markers. Violin plots in panel a) show the expression of MSC specific marker genes and b) expression of reported marker genes absent in MSCs in all cells (top panel), all cells split by replicate (middle panel), and all cells split by time-point (bottom panel).

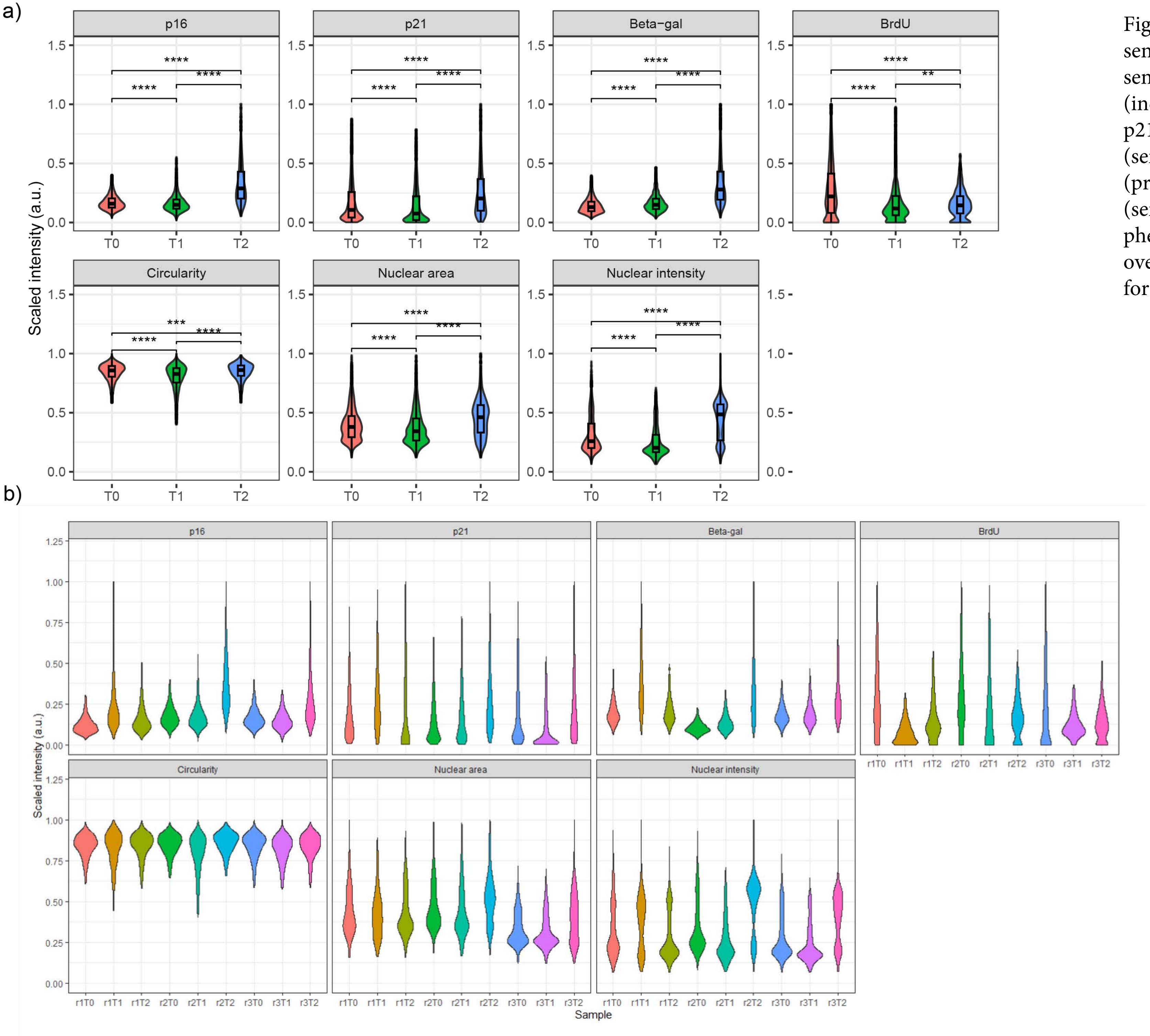

Figure S2. Validating the senescence phenotype through senescence specific markers (including DAPI (stain for DNA), p21 (senescence marker), p16 (senescence marker), BrdU (proliferation marker), SA-b-Gal (senescence marker) and phenotypic features, for (a) overall (p-value < 0.001) and (b) for individual replicates.

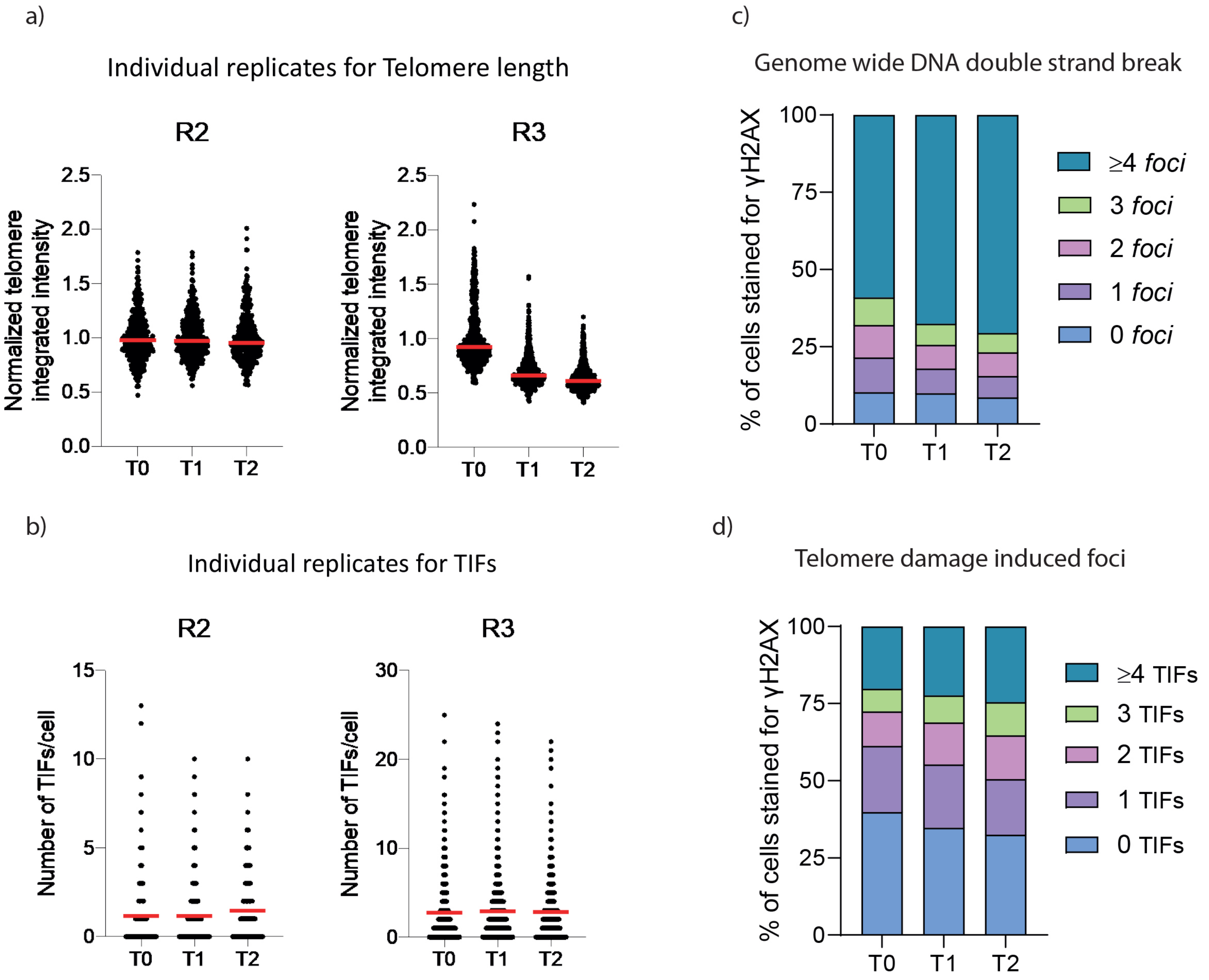

Figure S3. Telomere length and DNA damage analysis through replicative senesce in esMSCs in replicates 2 and 3. (a) Normalised telomere length across all time-points. (b) Telomere Dysfunction Induced Foci (TIF) analysis (R2: p-value = ns; R3: p-value < 0.001). The violin plot shows the number of cells that indicated DNA double strand break at the telomeres at each time-point. There is no significant difference between the three time-points (one-way ANOVA, p-value > 0.05 for all pairwise comparisons) (c) Genome wide DNA double strand break. The stack bar shows the percentage of cells that stained positive for γH2AX, genome wide. The number of cells with DNA damage increases from T0 to T2, however the extent of change is not statistically significant. (d) DNA double-strand break analysis at the telomeres. The stack bar shows the percentage of cells that stained positive for γH2AX, at the telomeres. Similar to the genome-wide analysis, we observe a gradual increase in number of cells from T0 to T2 but the difference in proportions are not statistically significant. *ns= non-significant.

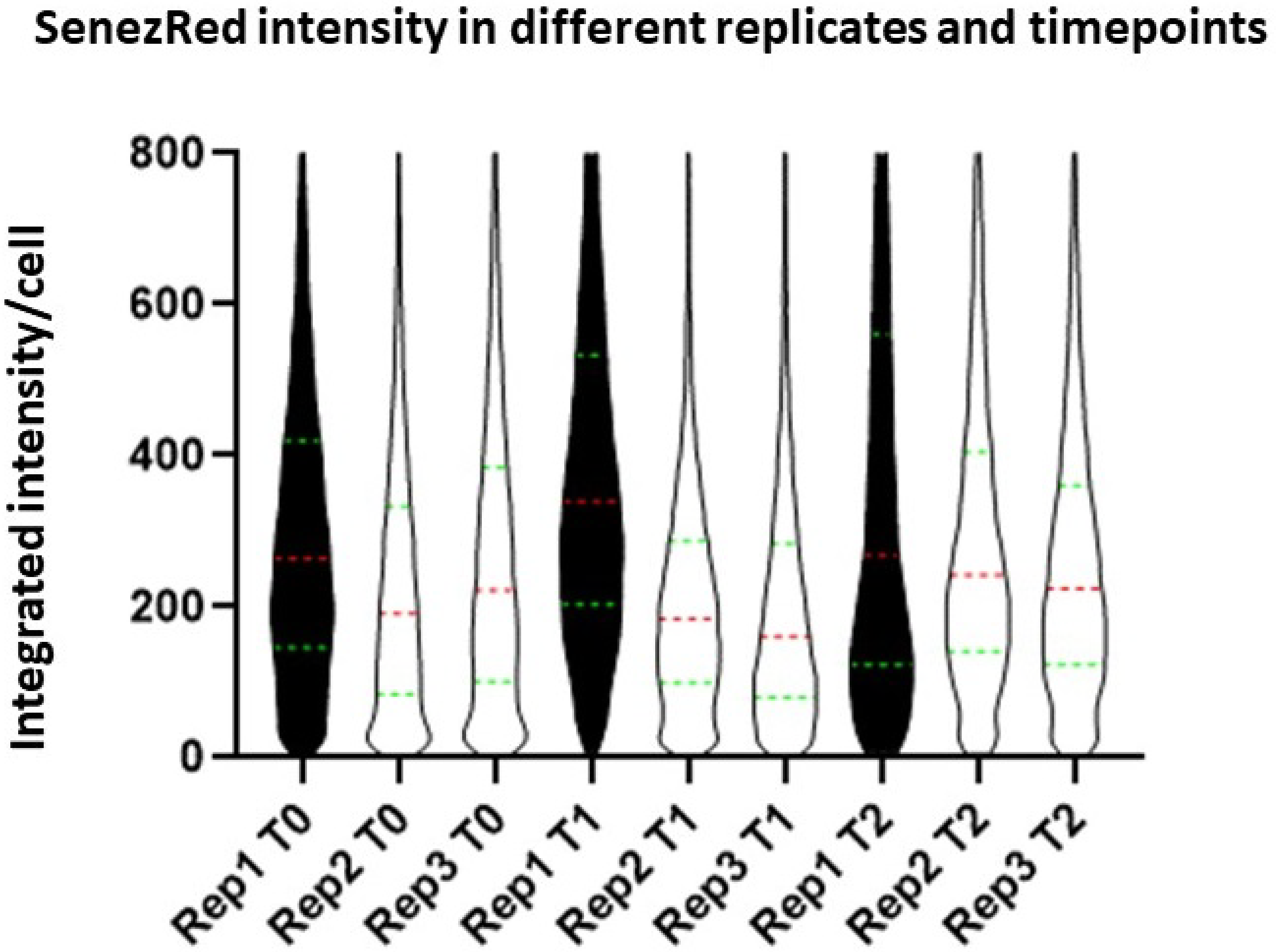

Figure S4. SenezRed staining show that there is no significant difference between different cells at different timepoints and replicates.

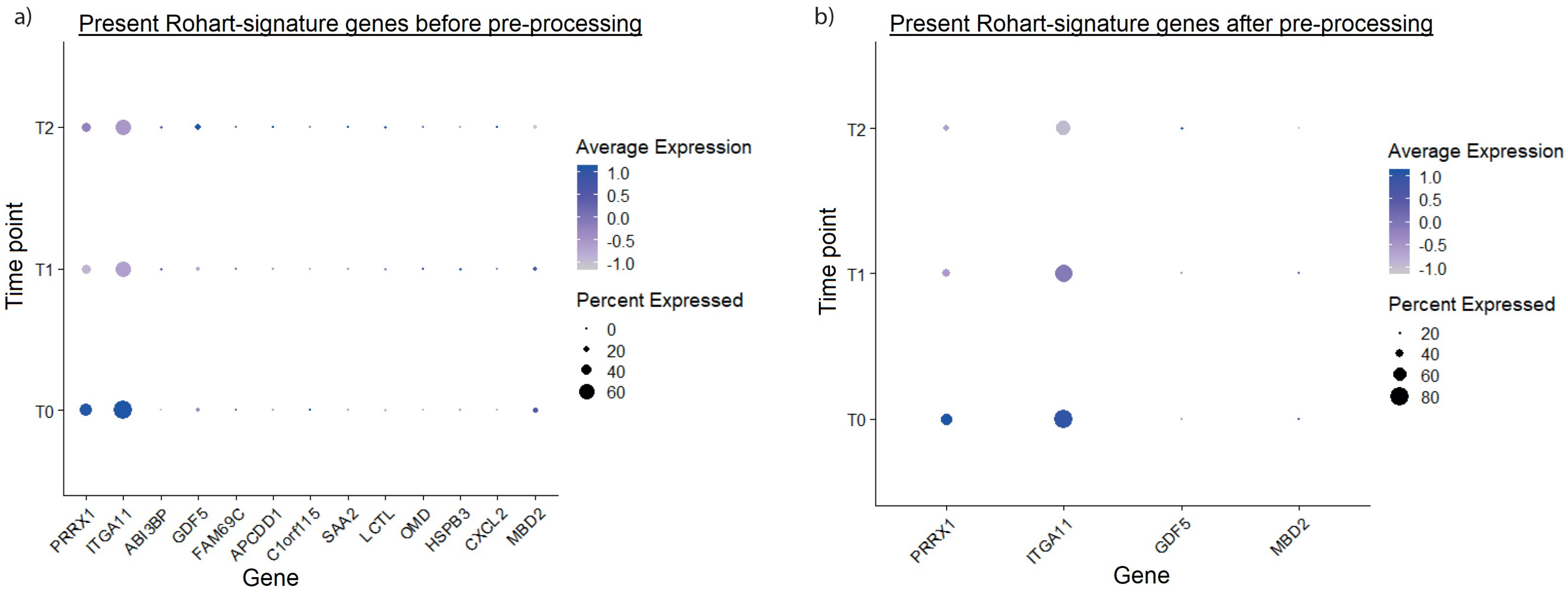

Figure S5. Evaluating the expression of Rohart MSC signature (a) before and (b) after pre-processing the data. Out of the 16 MSC-specific genes in the Rohart MSC signature, 13 genes were identified in our dataset prior to filtering for lowly expressed genes; and four genes remained after filtering (*i.e.* removing genes with less than 10% expression in all cells). More than 70% of the cells expressed ITGA11 and 41.14% of cells expressed PRRX1. This was followed by GDF5 and MBD2 with 19.36% and 14.78% of cells respectively.

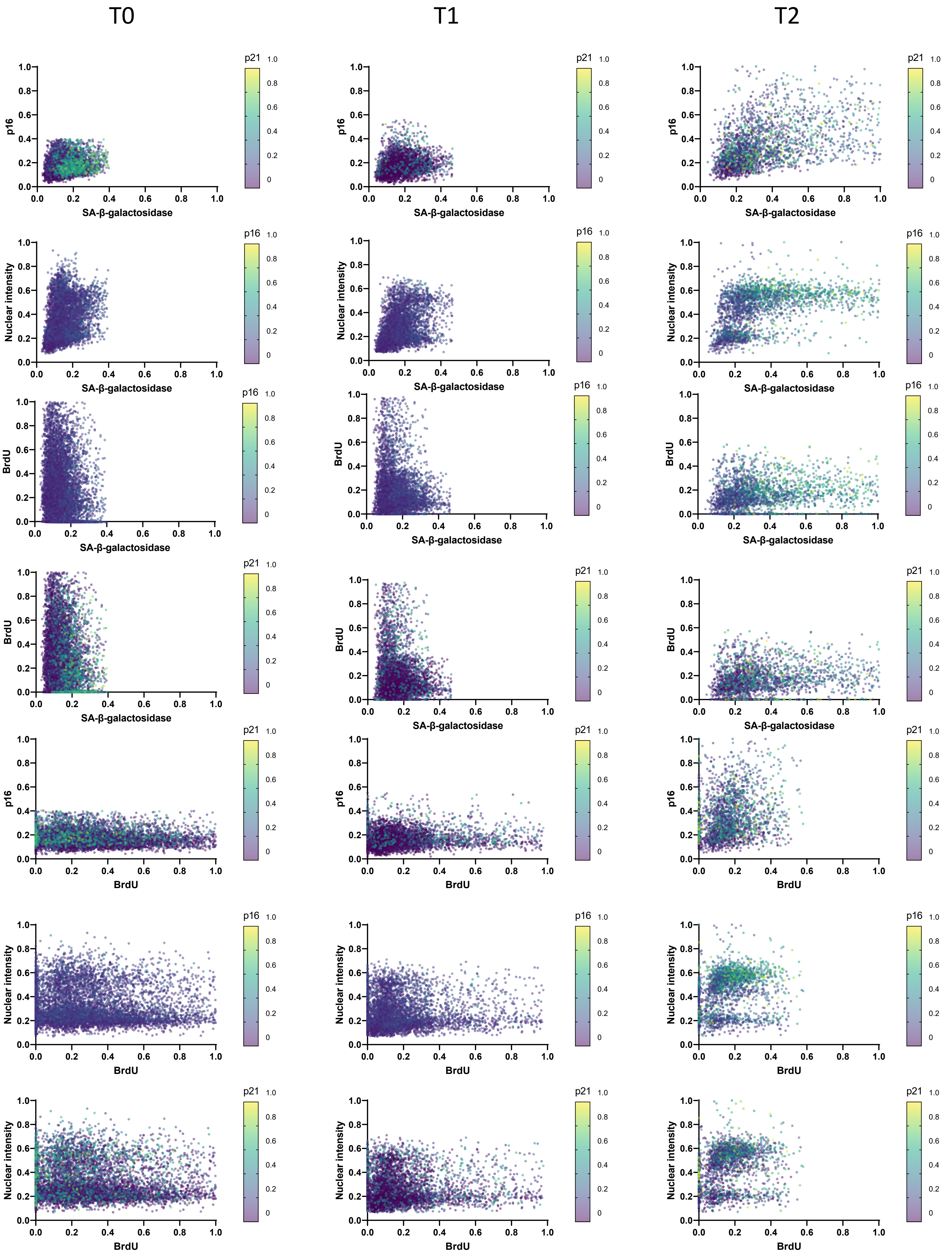

Figure S6a Correlation plot of nuclear p16 and p21, DNA content, SA-β-galactosidase activity and BrdU across T0, T1 and T2. Each dot represents a single cell.n >2,400.

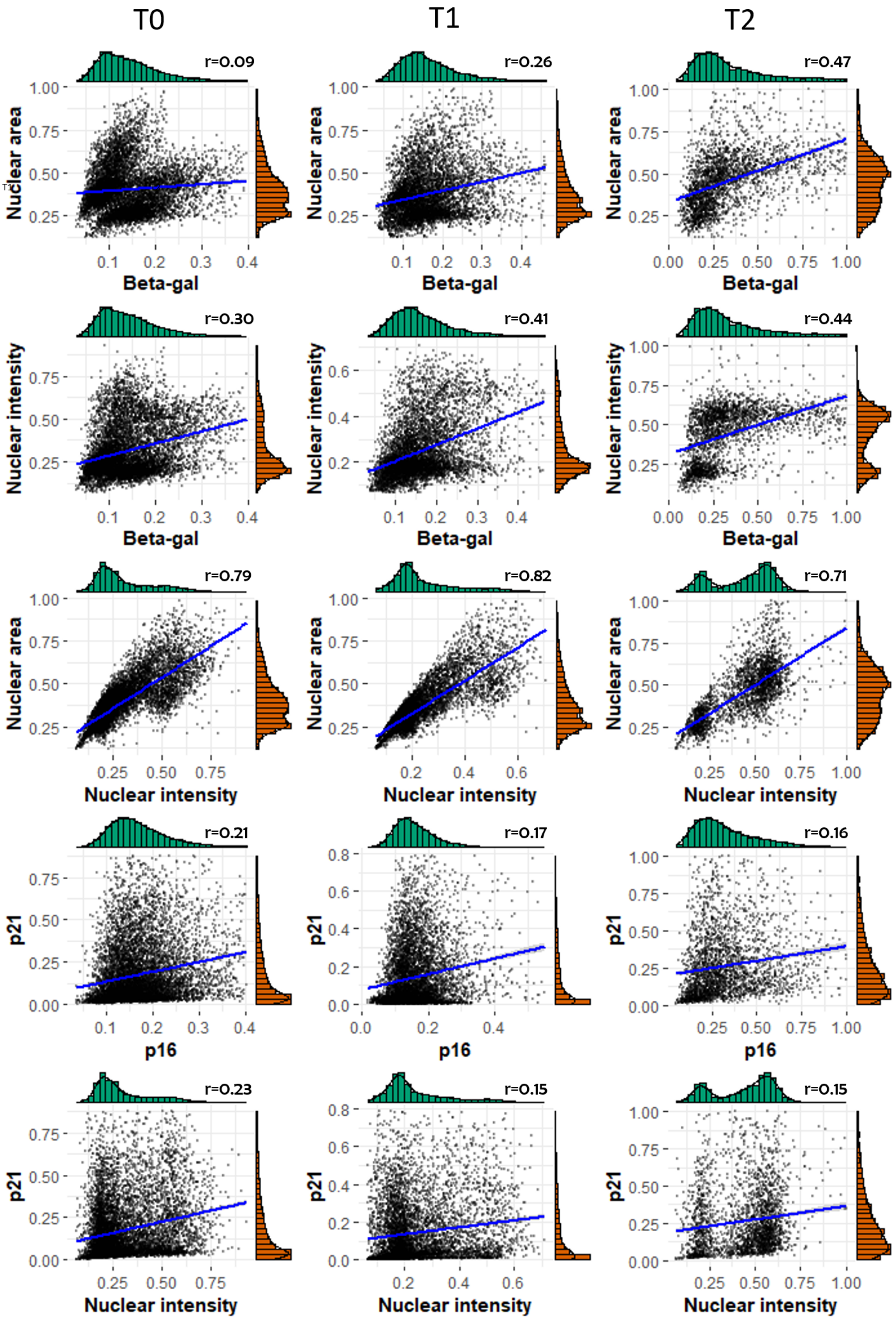

T0
T1
T2
r=0.09
r=0.26
r=0.47
r=0.30
r=0.41
r=0.44
r=0.79
r=0.82
r=0.71
r=0.21
r=0.17
r=0.16
r=0.23
r=0.15
r=0.15
Nuclear area
Nuclear intensity
p21
Beta-gal
Nuclear intensity
p16

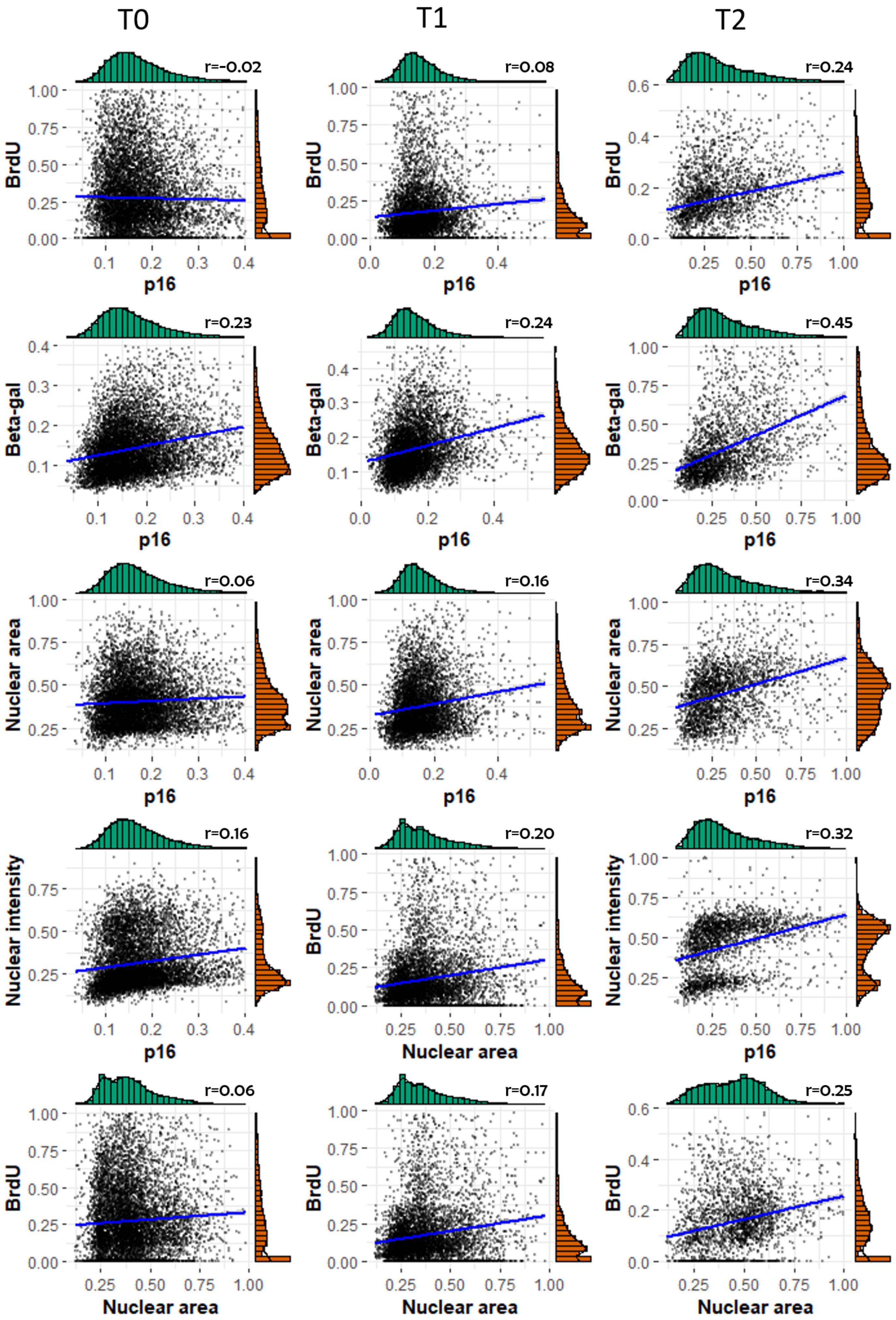

Figure S6b. Correlation analysis between senescence specific markers and phenotypic features. Pearson correlation was computed for each marker intensity (samples are pooled from replicate 2 and 3).

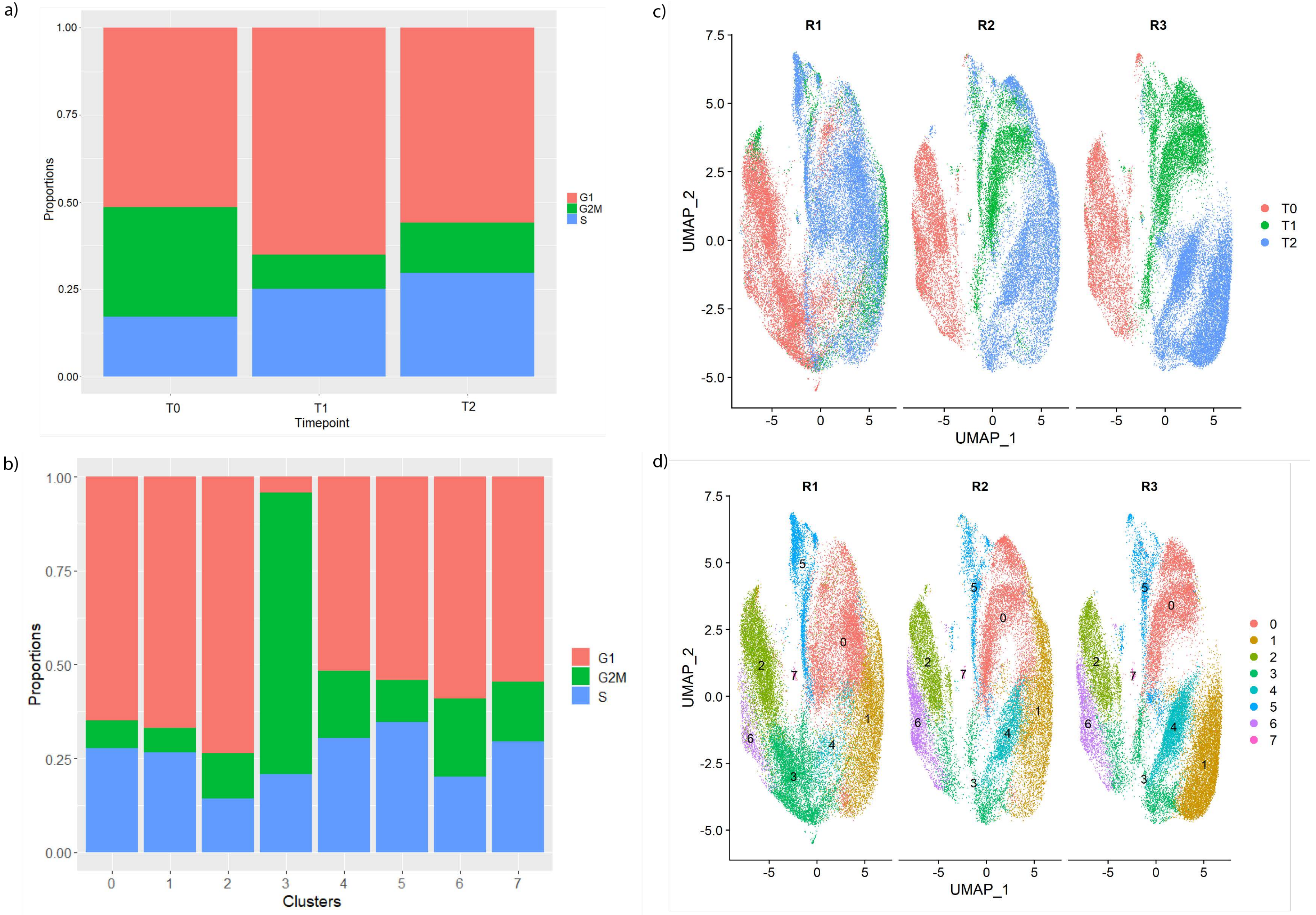

Figure S7. Proportion of cells in different cell cycle phases in different (a) timepoints and (b) clusters. (c-d) UMAP of cells in different replicates, color coded based on timepoint and cluster, respectively.

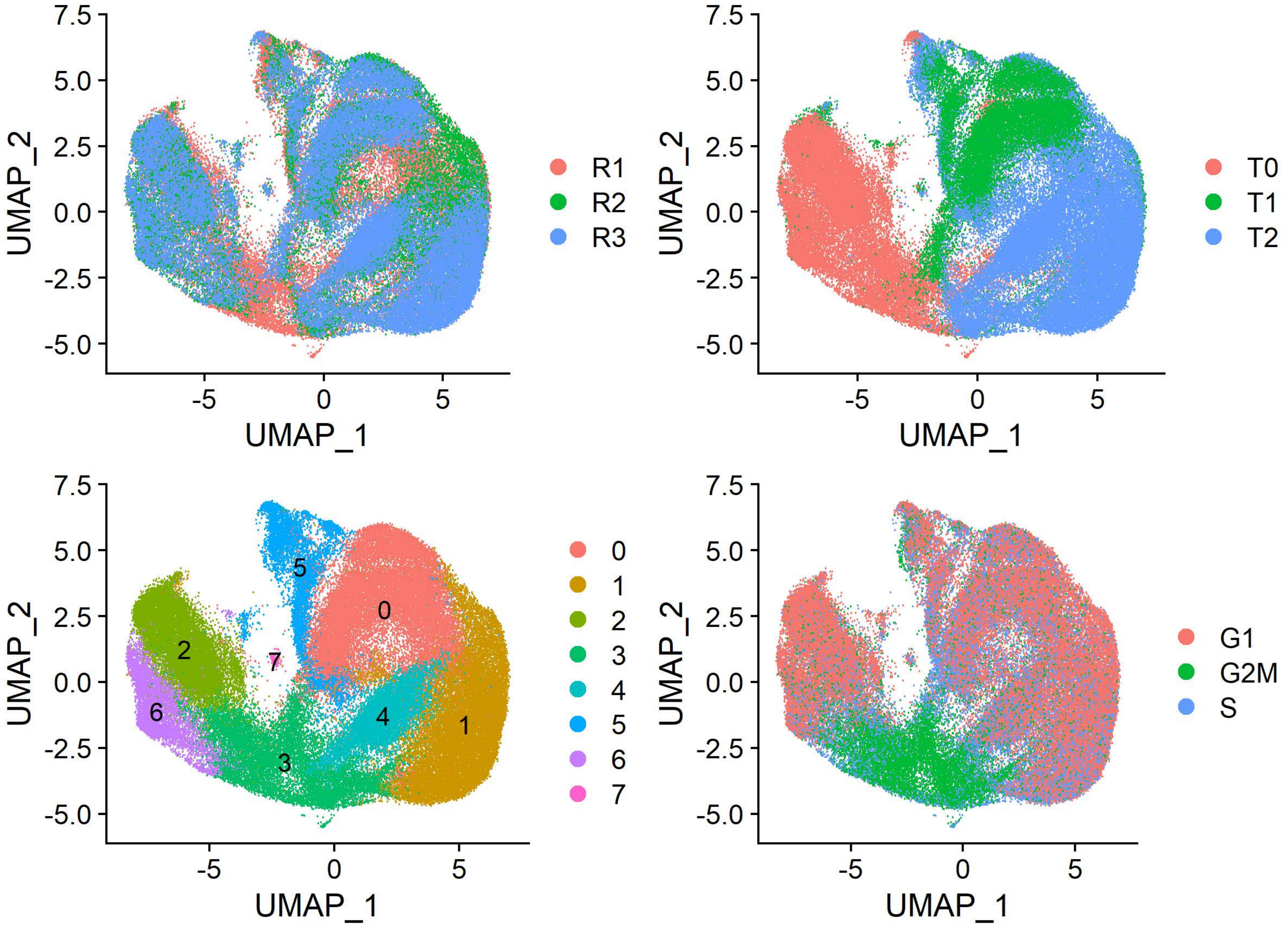

Figure S8. UMAP of cells, color coded by cluster and split by timepoint. Clusters 2, 6 and parts of cluster 3 comprise of T0 cells. Cluster 5 and 0 comprise of T1 cells, and proportion of cluster 3, 5 and 0, and majority of cluster 1 and 4 comprise of T2 cells.

Figure S9a. Top GO BP pathways (q-value < 0.05)
Full data in Table S4

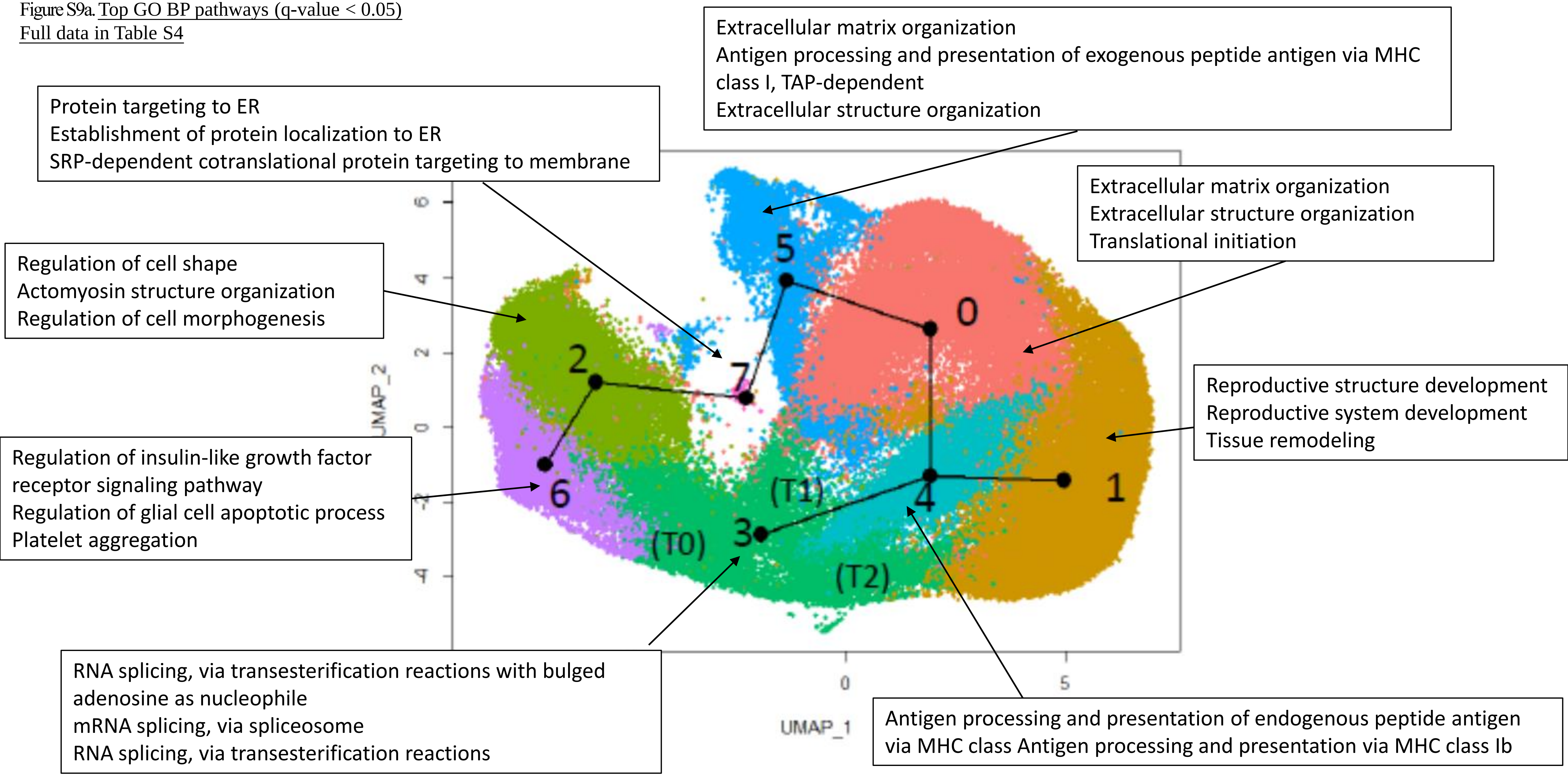

Figure S9b. Top marker genes based on the highest average fold change.
Full data in Table S5

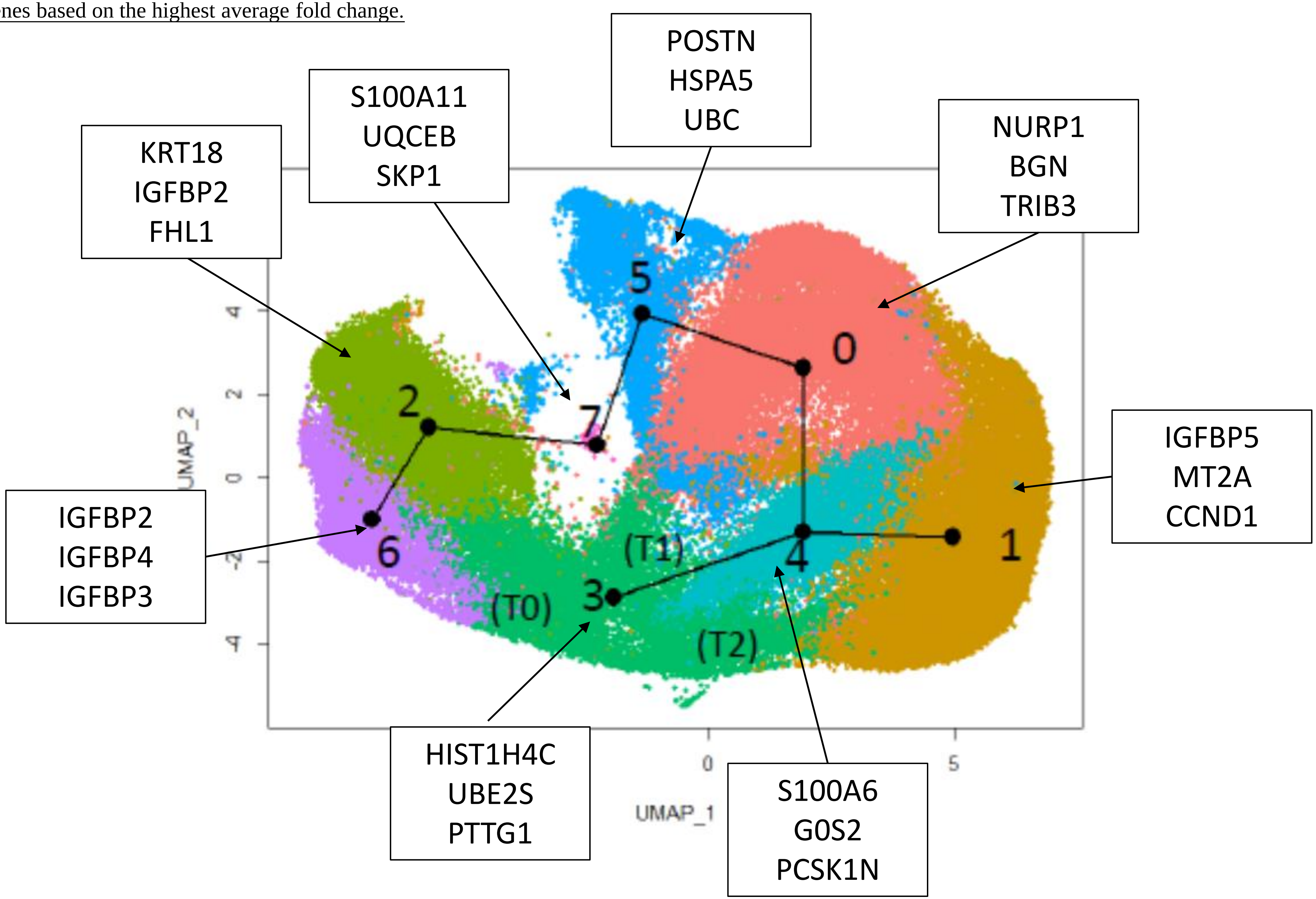

Figure S9c.Cell surface markers

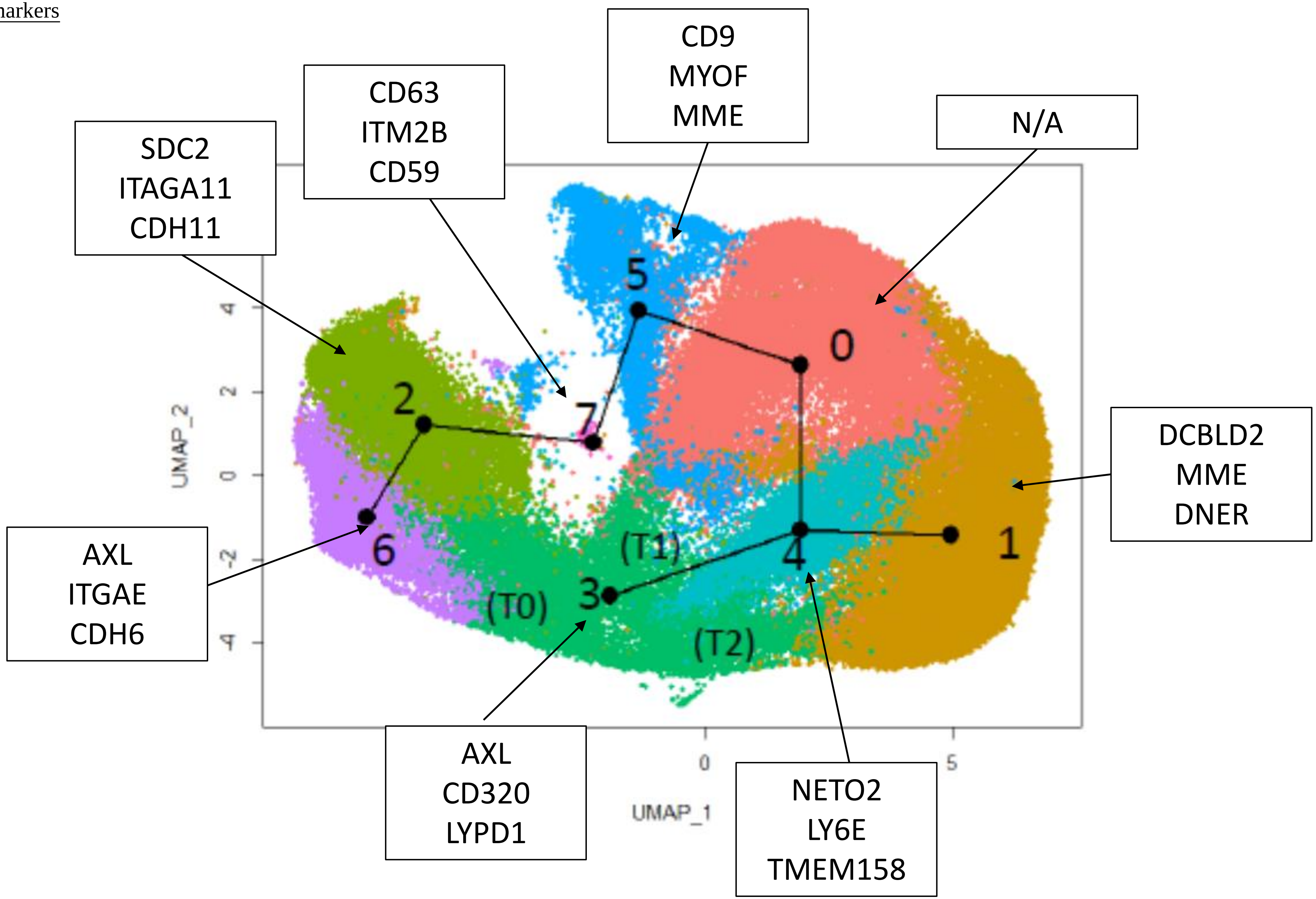

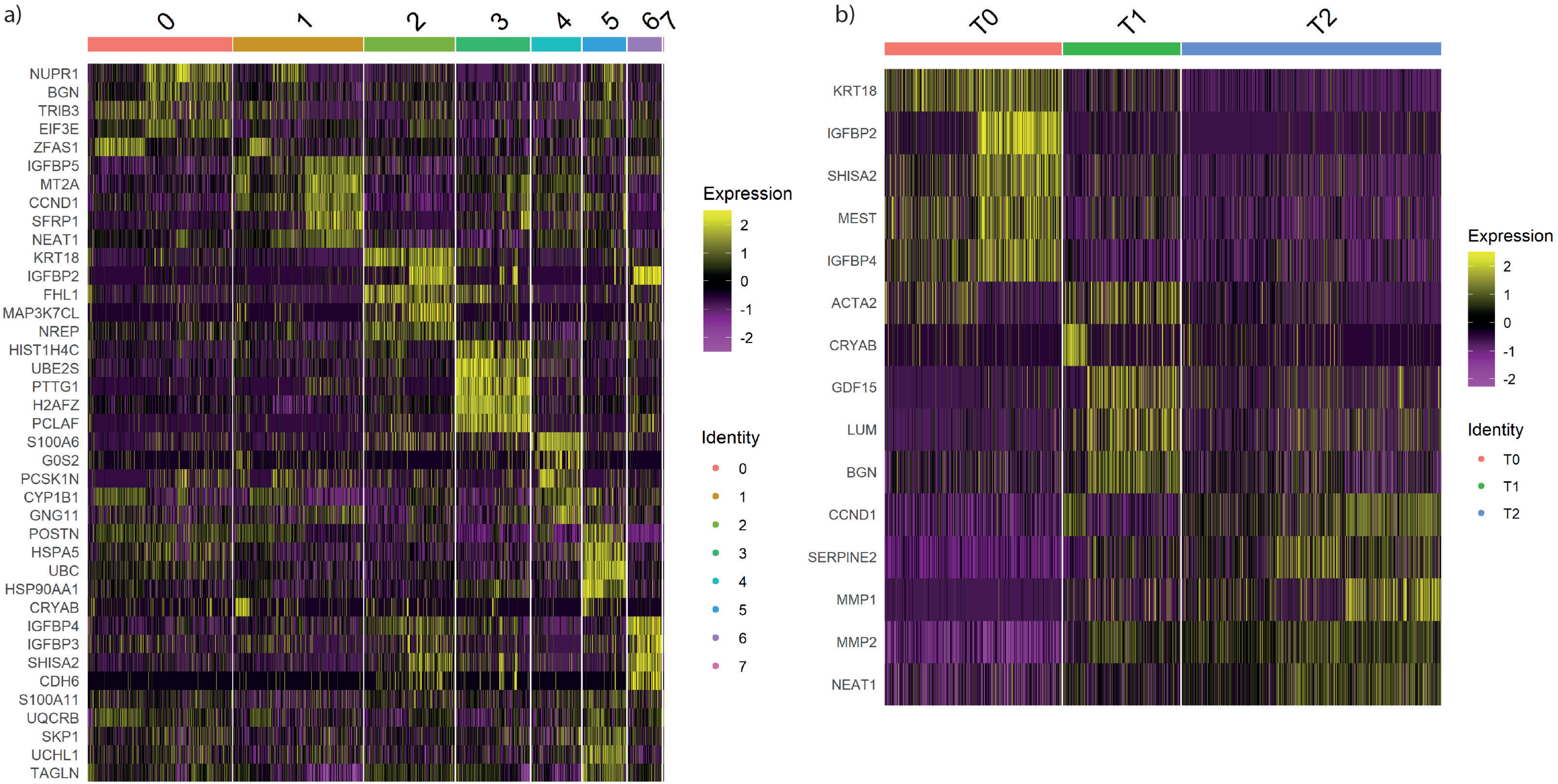

Figure S10. Normalised gene expression of top five marker genes for each (a) cluster and (b) time-point.

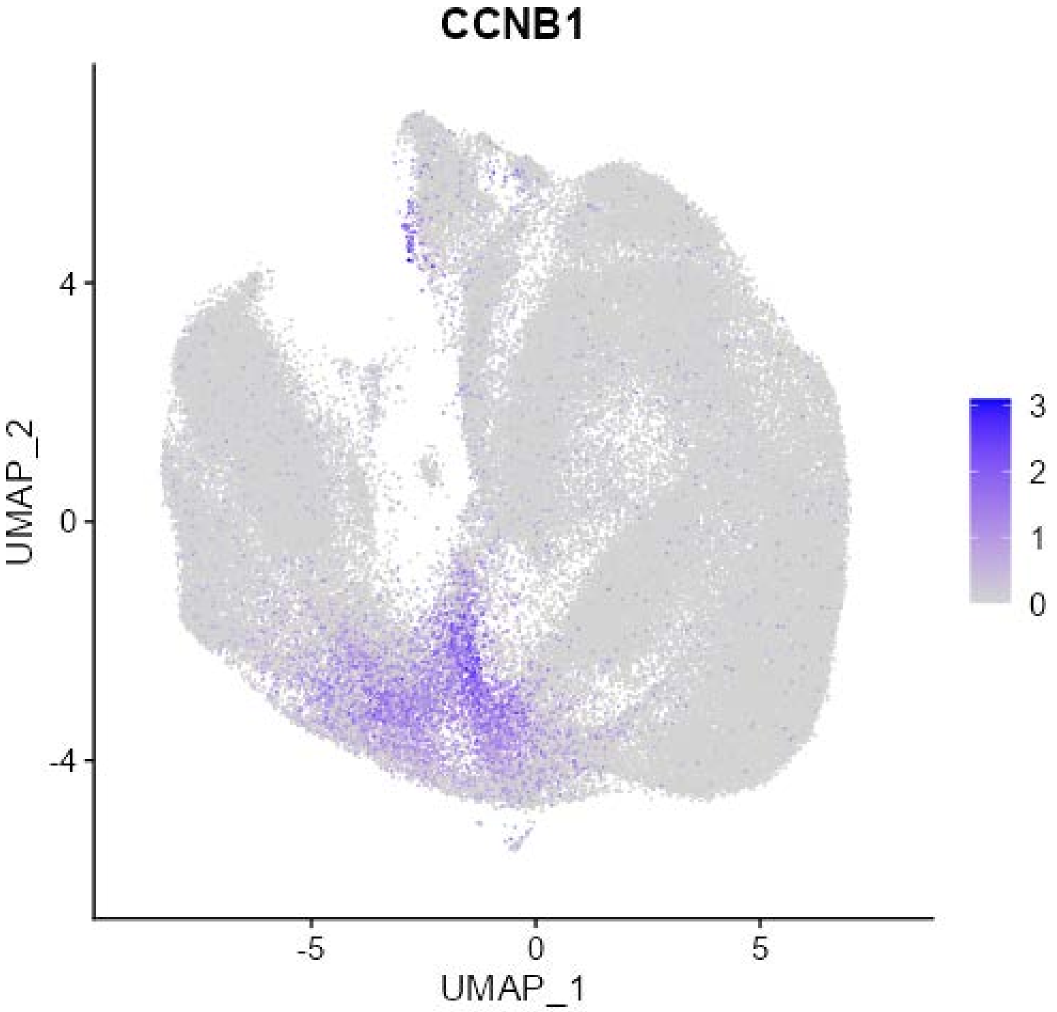

Figure S11. UMAP representing normalized expression of CCNB1. This gene has been implicated in p53 mediated permanent cell cycle arrest during senescence, which is highly expressed specifically in T2 subpopulation of cluster 3.

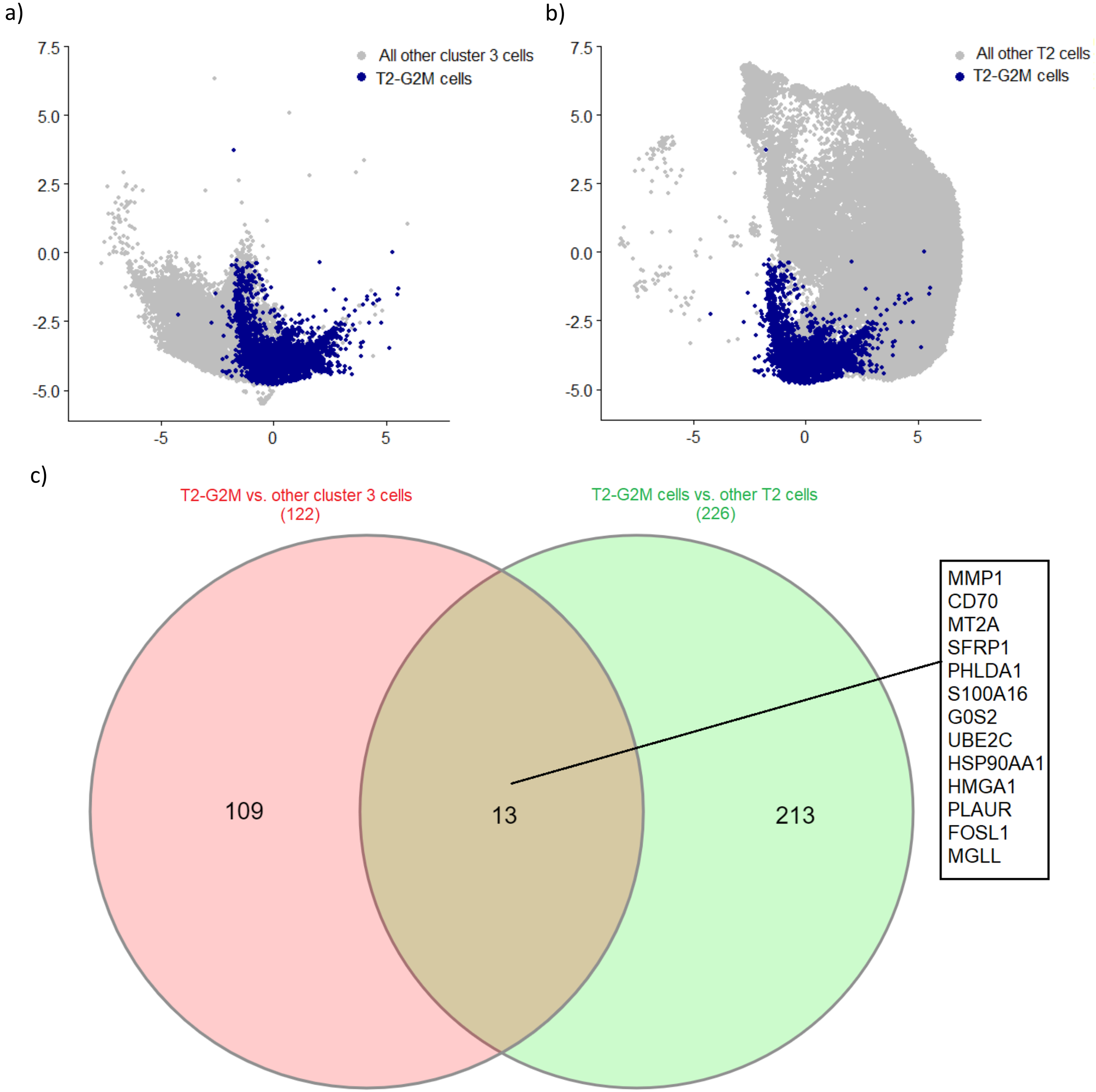

Figure S12. Differential expression analysis of T2-G2M cells (in blue) with (a) Proliferative esMSCs subtype-3 (other cells in cluster 3), and (b) other senescent cells (other T2 cells). Top panel: UMAP highlight cells in the comparison. Bottom panel: Significant DE genes (LogFC x > |0.25|, Bonferroni adjusted p-value<0.001). (c) Overlap between differentially expressed genes of T2-G2M cells with proliferative esMSC subtype-3 (other cells in cluster 3), and other senescent cells (other T2 cells). The Venn diagram shows the overlap between differentially expressed genes of T2-G2M cells with proliferative esMSCs subtype-3 (other cells in cluster 3), and other senescent cells (other T2 cells).

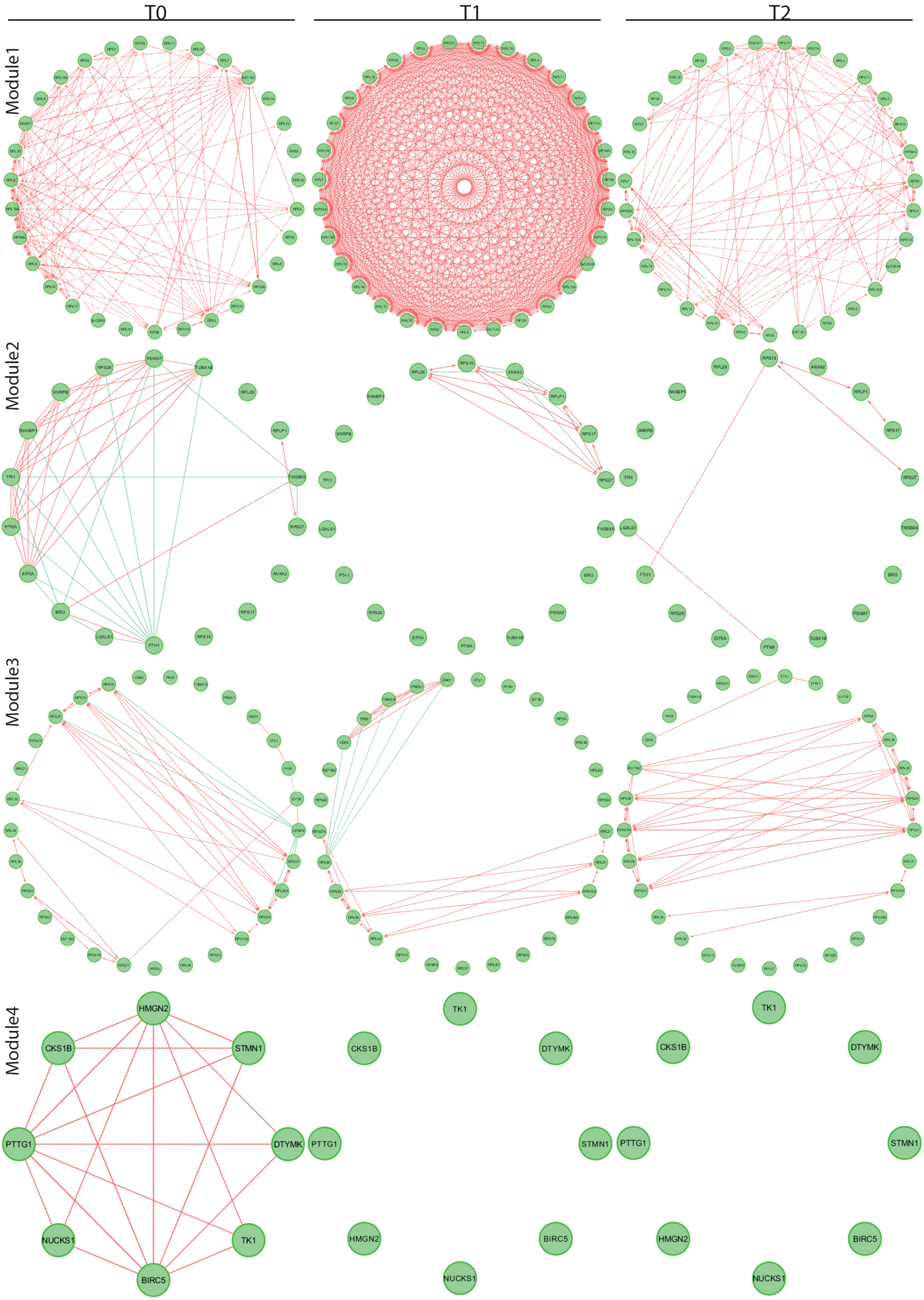
T0
T1
T2
Module1
Module2
Module3
Module4
PSMA7
RPS26
TUBA1B
SNRPB
RPL28
RANBP1
RPLP1
TPI1
TMSB4X
PTMA
RPS27
EIF5A
ANXA2
BRI3
RPS17
LGALS1
FTH1
RPS19
HMGN2
CKS1B
STMN1
PTTG1
DTYMK
NUCKS1
TK1
BIRC5

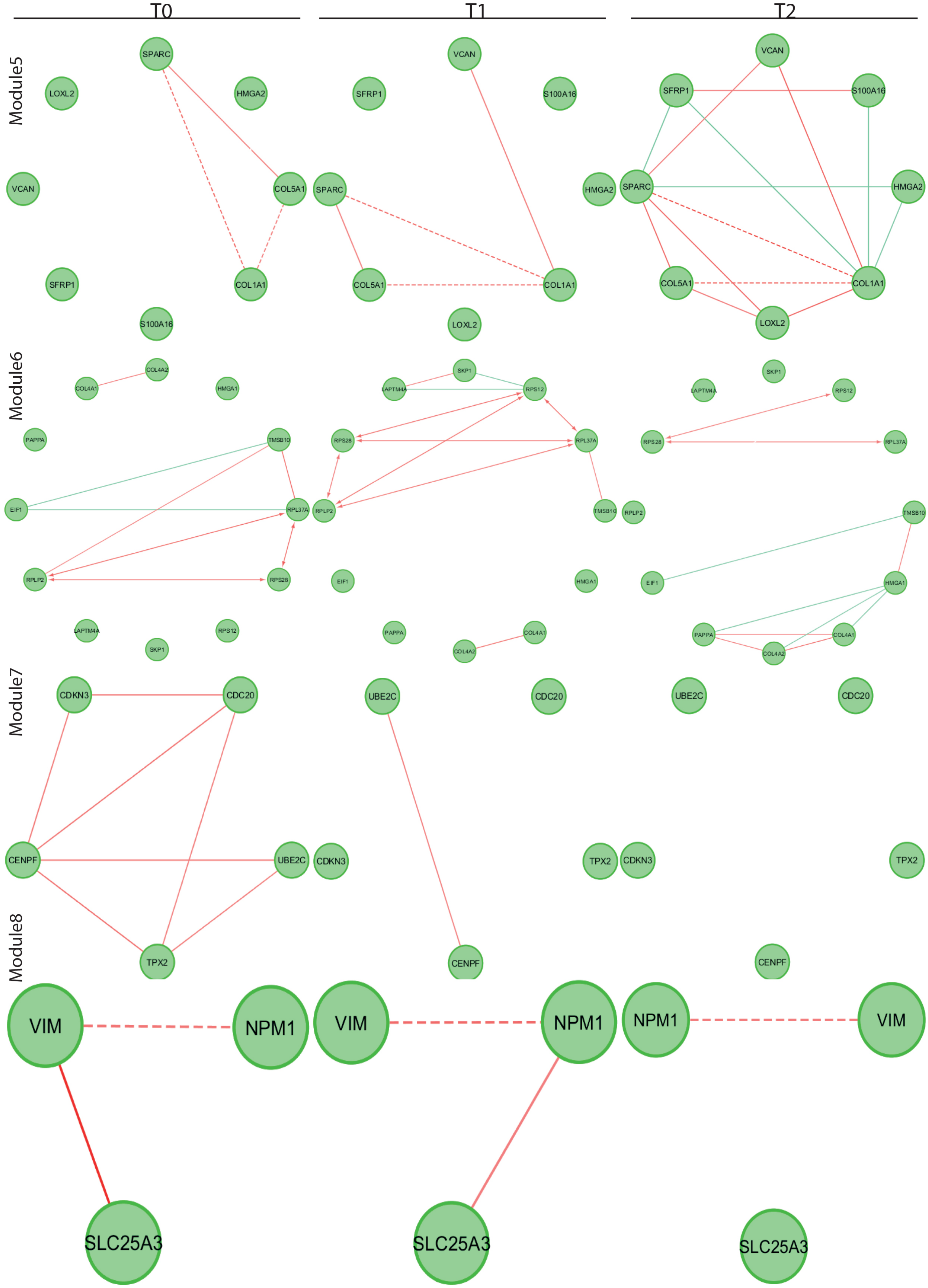

Figure S13. Functional interaction (FI) network analysis at each time-point. Nodes represent the genes in the PPI network (extracted via SCENT package) and edges represent the spearman-correlation computed from gene expression data. Highly connected modules were obtained via MCODE app in Cytoscape. Red and green edge colors represent positive and negative correlations, respectively. Edge types were extracted from ReactomeFIViz app in Cytoscape which has access to the Reactome pathway database. They show the mode of regulatory interaction: "->" for activating/catalyzing, "-|" for inhibition, "-" for FIs extracted from complexes or inputs, and "---" for predicted FIs.

Deconstructing replicative senescence heterogeneity of human mesenchymal stem cells at single cell resolution.

<u>Cluster specific characterisation of cell sub-populations reveals the different roles that esMSC states adopt to promote senescence.</u>

To gain insights into the biological processes that were enriched within the esMSC cell clusters, gene set over-representation analysis was performed using the marker genes specific to a cluster (see Methods).

Cluster 2 represents proliferative healthy esMSCs and its marker genes are mainly associated with proliferation and muscle development.

A large proportion of Cluster 2 (96.88%) is comprised of cells at T0, that is, the early or proliferate stage of the esMSCs (Fig. 3b). Marker genes of this cluster were significantly enriched for pathways associated with regulation of cell shape, epithelial to mesenchymal transition (EMT) and muscle cell differentiation (Table S2). Of note, these cells displayed high expression of *CSRP2*, a member of CSRP family encoding a group of short LIM domain proteins, which is a critical for cell-cycle progression, development and smooth muscle differentiation [1] (Tables 2, S2 and S3).

Cluster 6 represents proliferative healthy MSCs, with high expression of genes involved in insulin-like growth factor signalling.

Cluster 6 of T0 esMSCs were distinctly enriched for pathways associated with Insulin-like growth factor (IGFs) receptor signalling (Table S2). IGFs are known to play an important role in promoting proliferation, self-renewal and differentiation in MSCs [2]. Specifically, cluster 6 displayed high expression of IGF-binding proteins 2 and 4 (*IGFBP2* and *IGFBP4*) as compare to the other clusters (Fig. S10 and Table S3). Apart from mediating cell metabolism, development and growth, IGFBP4 plays an important role in promoting senescence in MSCs. It also inhibits osteoblast differentiation by inhibiting the potentiation effect of IGF-2 on bone morphogenetic protein-9 (*BMP-9*) in MSCs. IGFBP-2 is associated with keratocyte phenotype by increasing the expression of keratocan and *ALDH1A1* and decreasing α-smooth muscle actin [2, 3].

Cluster 5 represents cells undergoing metabolic stress, overlaps with SASP markers and is dominated by the cells from later stages of time-course.

Cluster 5 contains similar proportions of T1 (41.71%), and T2 (52.82%) cells (Fig. 3b). Similar high portions of T1 and T2 cells in this cluster indicate the transitional state of these esMSCs from a proliferative state into a senescent state. In this cluster, there is enrichment for genes related to metabolic stress regulation of G2M transition, reactive oxygen species (ROS), oxidative phosphorylation, and Glycolysis (Table S2), pathways previously implicated in metabolic changes that are implicated in promoting senescence [59-61] and ageing [62-65]; higher expression of tissue specific regulators (APOE: Adipogenic, COL1A1: Chondrogenic, THBS1: Osteogenic, ACTA2: Myogenic) and Mitochondrial NADH dehydrogenase subunit genes 2 and 3 (MT-ND2/3) that play an important role in the production of ROS [62]. Indeed, these cells from cluster 5 are computationally projected to transition into cluster 0, which contains roughly equal proportions of T1 cells (52.11%), and T2 cells (44.12%) (Fig. 3b).

Cluster 0 cells comprise of cells in the later stages of the time-course, corresponding to the pre-senescent state.

The majority of cluster 0 comprise of T1 cells (52.11%), followed by T2 cells (44.12%) (Fig. 3b). The difference in cell proportions is not significant which means T1 and T2 cells are roughly in equal proportions this cluster (FDR = 0.43).

Pathway over-representation analysis showed a significant enrichment for Apoptosis and p53 pathways (Table S2). Cells in this cluster displayed a high expression of TRIB3, an inhibitor of cell proliferation [66] that becomes upregulated in response to several forms of cellular stress [67] including oxidative ER stress and hypoxic stress (pathways that are all significantly enriched in cluster 5). We further noted strong expression of *NUPR1* in cluster 0 (Fig. S10 and Table S5), a gene involved in regulating resistance to micro-environmental stress, cell-cycle, apoptosis and DNA repair response [68] and that was previously found to promote K-ras induced senescence in the pancreas of mice [4].

Cluster 4 represents oncogenic associated senescence, majority of the cells from the T2 experiment design; oncogene and tumor suppressor marker genes.

A distinguishing feature about Cluster 4 is that its markers are enriched for oncogenes or tumor suppressor genes (23.48%), suggesting these correspond to cells undergoing oncogene-associated senescence (Fig. S10). Some of these markers include G_0-G_1 switch gene 2 (*G0S2*), a tumor suppressor gene associated with human dermal fibroblasts senescence, hematopoietic stem cell quiescence, adipocyte differentiation and cell-cycle withdrawal [5]. Another notable gene in cluster 4 is transcription elongation factor A protein-like 7 (*TCEAL7*), a gene that is known to regulate human telomerase reverse transcriptase (*hTERT*) expression and telomerase activity by inhibiting c-Myc pro-oncogene in cells that have activated the alternative lengthening of telomeres (ALT) mechanism. This is significant as more than 70% of mesenchymal tumours use the ALT pathway to maintain the telomere length and bypass replicative senescence [6, 7].

Cluster 1 represents SASP associated senescence, with marker genes as SASP factors, and SASP associated pathways.

Similar to other clusters, the number of SASP factors are upregulated in cluster 1. These include growth differentiation factor 15 (*GDF15*) [8], *THBS1* [9] and *MMP14* [10] (Fig. S10, Table S3). However, what makes this cluster "SASP-associated" is the exclusive enrichment of TGF-β signalling and inflammatory response, pathways with well-established roles in regulating cellular senescence [10-12]. Notably, there is a strong link between inflammatory response and senescence, as SASP includes inflammatory cytokines and chemokines [10, 13]. The marker genes in cluster 1 that are associated with the inflammatory pathway include *CCL2, CD70, CDKN1A, DCBLD2, EREG, HIF1A* and *MMP14* (Table S3). Moreover, SASP induces the production and expression of TGF-β, a growth factor known to induce and maintain a senescent phenotype and age-related pathological conditions [14].

Cluster 3 comprise of mix of proliferative and senescent cells that are in G2M cell cycle phase.

Pathways enriched in this cluster relate to cell cycle, nuclear division, and G2M check point, indicative of proliferative capabilities of these cells. Moreover, MYC-target and cellular senescence pathways highlight the senescent features of these cells (Table S2).

It is important to note that although more that 60% of the cells in this cluster belong to T0, approximately 11% and 26% belong to cells from T1 and T2, respectively (Figure 3b). More than 75% of this cluster are in the G2M cell cycle phase, indicating the proliferative capability of these cells. The top five cluster 3 specific markers are associated with cell cycle regulated anaphase-promoting complex. These include *UBE2S*, which is a cell cycle regulated ubiquitin ligase that controls progression through mitosis [15]. *PTTG1* is a protein coding gene which plays a central role in chromosome stability, by negatively regulating the transcriptional activity and related apoptosis activity of TP53 [16]. *PCLAF*, acts as a regulator of DNA repair during DNA replication and its expression peaks at G2M cell cycle phase [17] . From these results, we further characterised two sub-populations from Cluster 3 (*i.e.* T0 and T2) where each represents a distinct role of MSCs with respect to senescence. We performed two sets of differential expression analysis. First comparison was with Cluster3-T0 subpopulation with other T0 cells, and the second comparison involved Cluster 3-T2 subpopulation against the rest of cells from T2 (Figure S12).

Cluster 3-T0 sub-population are proliferative healthy esMSCs, with markers genes associated with proliferation.

In the T0 sub-cluster 3 the top DE genes (Table S4) in esMSC cells were enriched for genes related to regulation of G2M cell cycle progression. These included *UBE2S* and *PTTG1*, genes that are associated with cell-cycle regulation through the anaphase-promoting complex, and *CKS1B*, which is associated with cell proliferation through regulation of cyclin-dependant protein serine/threonine kinase activity [86].

Cluster 3-T2 subpopulation are cells that have potentially escaped senescence; with a mix of SASP factors and proliferation-specific marker genes.

We next examined the top significantly up-regulated DE genes in T2 cluster 3 (Fig. S12 and Table S4). This revealed that these cells show significantly increased expression of SASP factors (*MMP1*, *SERPINE2*, *MMP2*), as compare to the cluster 3 T0 subpopulation (Fig. 5a). They also displayed strong expression of *CCND1*, a well-established regulator of CDK kinases throughout the cell cycle, and a protein that specifically interacts and regulates CDK4/CDK6 that are required for cell cycle G1/S transition [18]. Compared to the rest of T2 cells in the dataset (Fig. 5b and Fig. S12), T2 subcluster 3 displays increased expression of *BIRC5*, an anti-apoptotic gene linked to G2M cell cycle phase, suggesting that the cells in this cluster have likely escaped cell cycle arrest or never entered cellular senescence in the first place [19]. The fact that this cluster also uniquely over-expresses *TPX2*, a gene that promotes chromosomal instability and escape of cell cycle arrest and senescence [20, 21].

1. Wang, S.-J., et al., *Cysteine and glycine-rich protein 2 (CSRP2) transcript levels correlate with leukemia relapse and leukemia-free survival in adults with B-cell acute lymphoblastic leukemia and normal cytogenetics*. Oncotarget, 2017. **8**(22): p. 35984-36000.

2. Youssef, A., D. Aboalola, and V.K.M. Han, *The Roles of Insulin-Like Growth Factors in Mesenchymal Stem Cell Niche.* Stem Cells International, 2017. **2017**: p. 9453108.
3. Sanada, F., et al., *IGF Binding Protein-5 Induces Cell Senescence*. Frontiers in Endocrinology, 2018. **9**(53).
4. Grasso, D., et al., *Pivotal Role of the Chromatin Protein Nupr1 in Kras-Induced Senescence and Transformation.* Scientific reports, 2015. **5**: p. 17549-17549.
5. Yim, C.Y., et al., *G0S2 Suppresses Oncogenic Transformation by Repressing a MYC-Regulated Transcriptional Program.* Cancer Research, 2016. **76**(5): p. 1204-1213.
6. Lafferty-Whyte, K., et al., *TCEAL7 inhibition of c-Myc activity in alternative lengthening of telomeres regulates hTERT expression.* Neoplasia (New York, N.Y.), 2010. **12**(5): p. 405-414.
7. Royle, Nicola J., et al., *The role of recombination in telomere length maintenance.* Biochemical Society Transactions, 2009. **37**(3): p. 589-595.
8. Guo, Y., et al., *Senescence-associated tissue microenvironment promotes colon cancer formation through the secretory factor GDF15.* Aging Cell, 2019. **18**(6): p. e13013.
9. Guillon, J., et al., *Regulation of senescence escape by TSP1 and CD47 following chemotherapy treatment.* Cell Death & Disease, 2019. **10**(3): p. 199.
10. Freund, A., et al., *Inflammatory networks during cellular senescence: causes and consequences.* Trends in Molecular Medicine, 2010. **16**(5): p. 238-246.
11. Kandhaya-Pillai, R., et al., *SMAD4 mutations and cross-talk between TGF-β/IFNγ signaling accelerate rates of DNA damage and cellular senescence, resulting in a segmental progeroid syndrome—the Myhre syndrome.* GeroScience, 2021. **43**(3): p. 1481-1496.
12. Ren, J.-L., et al., *Inflammatory signaling and cellular senescence.* Cellular Signalling, 2009. **21**(3): p. 378-383.
13. Lasry, A. and Y. Ben-Neriah, *Senescence-associated inflammatory responses: aging and cancer perspectives*. Trends in Immunology, 2015. **36**(4): p. 217-228.
14. Tominaga, K. and H.I. Suzuki, *TGF-β Signaling in Cellular Senescence and Aging-Related Pathology*. International journal of molecular sciences, 2019. **20**(20): p. 5002.
15. Brown, N.G., et al., *Dual RING E3 Architectures Regulate Multiubiquitination and Ubiquitin Chain Elongation by APC/C.* Cell, 2016. **165**(6): p. 1440-1453.
16. Bernal, J.A., et al., *Human securin interacts with p53 and modulates p53-mediated transcriptional activity and apoptosis*. Nat Genet, 2002. **32**(2): p. 306-11.
17. Emanuele, M.J., et al., *Proliferating cell nuclear antigen (PCNA)-associated KIAA0101/PAF15 protein is a cell cycle-regulated anaphase-promoting complex/cyclosome substrate.* Proc Natl Acad Sci U S A, 2011. **108**(24): p. 9845-50.
18. Day, P.J., et al., *Crystal structure of human CDK4 in complex with a D-type cyclin.* Proceedings of the National Academy of Sciences of the United States of America, 2009. **106**(11): p. 4166-4170.
19. Sumi, T., et al., *Survivin knockdown induces senescence in TTF-1-expressing, KRAS-mutant lung adenocarcinomas*. International journal of oncology, 2018. **53**(1): p. 33-46.
20. Chen, W.-S., et al., *Ran-dependent TPX2 activation promotes acentrosomal microtubule nucleation in neurons.* Scientific Reports, 2017. **7**(1): p. 42297.
21. Hsu, C.-W., et al., *Targeting TPX2 Suppresses the Tumorigenesis of Hepatocellular Carcinoma Cells Resulting in Arrested Mitotic Phase Progression and Increased Genomic Instability*. Journal of Cancer, 2017. **8**(8): p. 1378-1394.
